# Medicare Plan Switching and Hospice Care Among Decedents With Advanced Cancer

**DOI:** 10.1001/jamanetworkopen.2026.0755

**Published:** 2026-03-24

**Authors:** Xin Hu, Changchuan Jiang, Youngmin Kwon, Fangli Geng, Qinjin Fan, Kewei Sylvia Shi, Zhiyuan Zheng, Jingxuan Zhao, Joan L. Warren, K. Robin Yabroff, Xuesong Han

**Affiliations:** 1 Department of Radiation Oncology, School of Medicine, Emory University, Atlanta, Georgia; 2 Winship Cancer Institute, Emory University, Atlanta, Georgia; 3 Department of Health Policy and Management, Rollins School of Public Health, Emory University, Atlanta, Georgia; 4 Department of Internal Medicine, UT Southwestern Medical Center, Dallas, Texas; 5 Department of Health Policy, Vanderbilt University Medical Center, Nashville, Tennessee; 6 Department of Health Services, Policy and Practice, Brown University School of Public Health, Providence, Rhode Island; 7 American Cancer Society, Atlanta, Georgia

## Abstract

**Importance:**

Hospice is central to end-of-life (EOL) care for patients with advanced cancers and is an excluded benefit under Medicare Advantage (MA), with coverage instead provided by traditional Medicare (TM). With growing MA penetration, more beneficiaries also switch between MA and TM for financial protection and physician access considerations, although less is known about how different Medicare programs and plan switching behaviors affect EOL care for patients with advanced cancers.

**Objective:**

To evaluate hospice utilization and places of hospice care by Medicare plan switching patterns.

**Design, Setting, and Participants:**

This retrospective cohort study used Surveillance, Epidemiology, and End Results (SEER) Medicare data to identify beneficiaries aged ≥66 years diagnosed with distant-stage female breast, colorectal, lung, pancreatic, or prostate cancers from 2010 to 2019 who died by 2020. Beneficiaries were followed for up to 1 year before death. Data were analyzed from August 1, 2024, to December 14, 2025.

**Exposure:**

Plan switching patterns classified as continuous MA, continuous TM, MA to TM, TM to MA, and other (ie, multiple switches).

**Main Outcomes and Measures:**

Main outcomes were hospice enrollment in the last year of life and within 3 days of death, total hospice length of stay, and place of last hospice stay (home, nursing home, hospice facility, inpatient facility, or other) using multivariable regressions.

**Results:**

The sample included 196 536 decedents (46.5% female, 49.2% aged 66-74 years). Plan switching was infrequent (1.5% TM to MA; 1.8% MA to TM). Those who switched plans were more likely to be members of racial and ethnic minority groups and dual Medicare-Medicaid enrollees. Hospice enrollment was highest for those with continuous MA (74.8%), followed by those who switched from TM to MA (69.0%), those with continuous TM (68.5%), and those who switched from MA to TM (66.4%). Continuous MA beneficiaries had longer hospice stays than continuous TM beneficiaries (48.3 vs 43.8 days). Compared with continuous TM, continuous MA beneficiaries were more likely to receive hospice at home (1.93 percentage points [pp]; 95% CI, 1.40-2.45 pp; *P* < .001), while those who switched from MA to TM were more likely to receive hospice in nursing homes (2.45 pp; 95% CI, 1.26-3.63 pp; *P* < .001), particularly among dual Medicare-Medicaid enrollees (6.01 pp; 95% CI, 2.80 to 9.21 pp; *P* < .001).

**Conclusions and Relevance:**

In this cohort study of Medicare decedents with advanced cancers, continuous MA enrollees were most likely to receive hospice at home, while those who switched from MA to TM more frequently received hospice care in nursing homes. Plan switching near the EOL may reflect access barriers, highlighting the importance of addressing care coordination to improve EOL care.

## Key Points

**Question:**

What are the hospice utilization implications for Medicare decedents with advanced cancers continuously enrolled in Medicare Advantage (MA), traditional Medicare (TM) or who switch plans in the last year of life?

**Findings:**

In this cohort study among 196 536 beneficiaries who died in 2010-2020, continuous MA enrollees had 6.93 percentage points higher probability of hospice use and 5.13 days longer stay than those with continuous TM. Continuous MA enrollees more often received hospice care at home, while those who switched from MA to TM more often received hospice care in nursing homes.

**Meaning:**

Results of this study suggest greater hospice use among MA decedents, which may improve end-of-life care; those who switched from MA to TM may represent a subgroup with greater nursing home needs and access barriers under MA.

## Teaser

This cohort study reports on differences in hospice use between Medicare decedents with advanced cancers who were continuously enrolled in Medicare Advantage, traditional Medicare, or who switch plans in the last year of life.

## Introduction

Hospice is a specialized, supportive, comfort-focused care model for people with serious illness near the end of life (EOL), such as those with advanced cancers. It prioritizes relief of pain and other symptoms, psychosocial and spiritual support for patients and families, and alignment of care with patient goals.^1,2^ Early hospice enrollment has been associated with improved quality of life,^3^ reduced unnecessary and burdensome care,^4^ and a greater likelihood of dying in the preferred setting, often at home.^5,6^ Despite these benefits, nearly one-third of patients with advanced cancer do not enroll in hospice before death.^7^

Medicare is the largest payer for both cancer treatment and hospice in the United States.^8,9^ A growing share of Medicare beneficiaries are enrolled in Medicare Advantage (MA), privately managed alternatives to traditional Medicare (TM).^10^ While MA plans manage most Medicare-covered services, hospice has historically been carved out of MA.^11,12^ In other words, when MA beneficiaries elect hospice, responsibility shifts to TM for hospice-related services, while MA retains responsibilities to manage or coordinate other services (although minimal) near the EOL. This carve-out arrangement gives MA plans financial incentives to encourage early hospice enrollment to offset high costs of intensive cancer treatments but also creates misaligned accountability for care coordination during hospice stay. Consistent with this dynamic, MA decedents are more likely to enroll in hospice than those in TM.^11^

At the same time, switching between MA and TM has become increasingly common.^13^ Importantly, patients newly diagnosed with cancer are more likely to switch from MA to TM than the general Medicare population.^14^ Although the underlying reasons for plan switching in patients with cancer are not fully understood, prior studies suggest potential challenges accessing oncologists and other specialists, as well as utilization management practices that contribute to unmet care needs and subsequent plan switching.^15,16,17^ For example, limited MA coverage of National Cancer Institute (NCI)–designated cancer centers^16^ may prompt patients to switch plans to access clinical trials and novel therapies, potentially prolonging active treatment and delaying hospice transition; narrower skilled nursing facility (SNF) networks and prior authorization under MA may prompt patients to move to TM for broader access after hospitalizations related to cancer treatment or complications, which can affect downstream hospice care, including greater reliance on certain care settings such as nursing homes. Conversely, continuous MA enrollment may facilitate earlier more coordinated hospice referral, potentially explaining higher enrollment and longer stays.^11^

Despite these clinical implications, little is known about whether beneficiaries who switch between MA and TM during the last year of life experience different hospice utilization. This evidence gap is especially salient amid recent efforts to integrate hospice into MA through the Value-Based Insurance Design model, a model discontinued by the Centers for Medicare and Medicaid Services in 2024.^18^ Under either arrangement, MA plans retain incentives to manage utilization near the EOL, yet it remains unclear how these incentives intersect with EOL care quality, particularly for individuals who switch plans close to death. Using the population-based Surveillance, Epidemiology, and End Results (SEER) Medicare data, we examined hospice enrollment, timing of enrollment, and place of hospice care among beneficiaries with distant-stage cancers by plan switching patterns during the last year of life.

## Methods

### Study Design and Sample

We conducted a retrospective cohort study of Medicare beneficiaries newly diagnosed with distant-stage female breast cancer, colorectal cancer, non–small cell lung cancer (NSCLC), small cell lung cancer (SCLC), pancreatic cancer, or prostate cancer from 2010 to 2019 who died by 2020 using the linked SEER Medicare data.^19^ SEER registries cover approximately 48% of the US population,^20,21^ and provide information on patients’ cancer history, demographic characteristics, and neighborhood-level characteristics. Medicare administrative data, including Medicare Beneficiary Summary File and claim files, capture monthly enrollment information (eg, Medicare program type) and health care utilization (eg, hospice enrollment).

We used *International Classification of Diseases for Oncology, Third Edition* (*ICD-O-3*) codes and histology codes to identify patients with the aforementioned 6 types of cancer^22^ (eTable 1 in Supplement 1). We selected the first distant-stage diagnosis among individuals aged 66 years and older, with known diagnosis month and not diagnosed on autopsy or death certificate. Patients had to have 3 months or greater survival for sufficient time to measure plan switching. We defined the index date as 1 year prior to death or date of diagnosis for those who survived less than 1 year; patients had to have continuous parts A/B coverage with or without part C (ie, managed care) from the index date to death (eFigure 1 in Supplement 1). This study was deemed exempt under Common Rule (45 CFR 46, subpart A) by the Morehouse School of Medicine Institutional Review Board; informed consent was not required. We followed the Strengthening the Reporting of Observational Studies in Epidemiology (STROBE) reporting guideline.

### Measures

#### Medicare Plan Switching

We used Medicare monthly enrollment indicators for up to 1 year before death to classify beneficiaries’ plan switching patterns: (1) continuous TM, (2) continuous MA, (3) switching from MA to TM, (4) switching from TM to MA, and (5) other switching patterns (ie, multiple switches). Of note, for patients who survived less than 12 months (eg, 3-month survival), plan switching patterns were assessed within the window from the date of cancer diagnosis to month of death (ie, 3-month window). Because Medicare open enrollment occurs annually,^13,23^ we conducted sensitivity analyses restricted to patients surviving 12 months or longer to allow plan switching pattern classification within a 12-month window. eFigure 2 in Supplement 1 illustrates plan switching patterns for patients with varying length of survival.

#### Hospice Utilization and Place of Last Hospice Care

Outcomes included hospice enrollment in the last year of life, late hospice enrollment (first hospice claim within 3 days of death among hospice users), total hospice length of stay, and site of last hospice service (home, nursing home, hospice facility, inpatient facility, or other [long-term care hospital; inpatient psychiatric facility; place not otherwise specified; hospice residential facility]). Hospice enrollment was extracted from the hospice fee-for-service claim file. We also evaluated late hospice enrollment and total hospice length of stay as potential quality indicators.^24,25^ The site of hospice service was identified using place-specific Healthcare Common Procedure Coding System codes (Q5001-Q5010; eTable 2 in Supplement 1) among beneficiaries with any hospice enrollment.^26^ Notably, as beneficiaries may transition to different sites of care during a hospice stay, we identified the Healthcare Common Procedure Coding System code shown in the last line of the last hospice claim.

#### Study Covariates

Patient baseline socioeconomic characteristics abstracted from medical records by cancer registrars at the time of diagnosis included age at diagnosis, sex, race and ethnicity (abstracted from medical records by cancer registrars at the time of diagnosis as Hispanic, non-Hispanic Black, non-Hispanic White, and other or unknown; the other group includes American Indian or Alaska Native, Asian or Other Pacific Islander, and other unspecified), and marital status. Census tract of residence at cancer diagnosis was used to identify metropolitan residency and quintiles of socioeconomic status (Yost Socioeconomic Index^27^). Clinical characteristics of cancer type and diagnosis year were available from SEER; dual Medicaid-Medicare eligibility was classified into never dual, gain dual, lose dual, always dually enrolled, and other patterns using the monthly dual eligibility code from the Medicare Beneficiary Summary File.^28^ We also measured county-level MA penetration rate as a proxy for market structure, which may confound plan enrollment and switching, as well as hospice enrollment.^12^

### Statistical Analysis

Sample characteristics by Medicare plan switching patterns were compared using χ^2^ tests. We described the distribution of hospice utilization and place of last hospice care by plan switching patterns, and conducted multivariable regression models to examine the differences in hospice utilization and place of hospice care by plan switching patterns, adjusted for all patient characteristics. Logistic models were used for any hospice utilization and late hospice enrollment, Poisson distribution was used for total hospice length of stay, and a multinomial model was used for place of last hospice care. Postestimation margins were used to estimate the adjusted differences in the probability of hospice use and a specific place of hospice care (eg, home vs all other places of hospice care) and adjusted differences in days for hospice length of stay.

We conducted sensitivity analyses restricted to patients surviving 12 or more months to address selection bias driven by prognosis and bias from underestimation of plan switching, given that the open enrollment period for plan switching occurs once per year.^13,23^ We also stratified the analysis by dual Medicare and Medicaid enrollment, given programmatic differences in the flexibility of plan switching for those with and without Medicaid coverage and the potential that Medicaid coverage of nursing home room and board may be associated with place of hospice care.^29,30^ Further, to address potential confounding of comorbidities, we examined a subgroup diagnosed in 2016 to 2019 for whom TM claims and MA encounter data were available to measure claim-based NCI comorbidity index in the year prior to diagnosis.^31^ Regressions with and without adjusting for comorbidity were conducted. Last, considering SNF utilization management and network restrictions under MA may prompt plan switching among beneficiaries with postacute care needs and affect choice of hospice site, we used a 2016-2019 subcohort during which SNF data files are available to describe switching patterns by SNF use after cancer diagnosis and estimated associations between plan switching and hospice outcomes with and without adjusting for SNF stay. Data analyses were performed using SAS version 9.4 (SAS Institute) and Stata software version 18.0 (StataCorp). Statistical significance was evaluated at the *P* < .05 threshold using 2-sided tests.

## Results

Among a total of 196 536 patients included in the analysis (eFigure 1 in Supplement 1), 46.5% were female, 53.5% were male, and 49.2% were aged 66 to 74 years. There were 10 873 (5.5%) with female breast cancer, 29 162 (14.8%) with colorectal cancer, 95 557 (48.6%) with NSCLC, 21 357 (10.9%) with SCLC, 21 564 (11.0%) with pancreatic cancer, and 18 023 (9.2%) with prostate cancer. Medicare plan switching was relatively infrequent, with 1.5% (2861) switching from TM to MA and 1.8% (3534) switching from MA to TM. Beneficiaries who switched from MA to TM were more likely to be younger (66-74 years: 53.8% vs 49.0%), from racial or ethnic minority groups (non-Hispanic Black: 16.2% vs 9.0%), and reside in areas with lower socioeconomic status (ie, Yost quintile 1: 20.1% vs 16.2%) than beneficiaries with continuous TM; similar differences were seen for beneficiaries who switched from TM to MA compared with beneficiaries with continuous TM. In particular, there was a higher proportion of always dually enrolled beneficiaries among those who switched from TM to MA (18.3%) and from MA to TM (22.2%) than those with continuous TM (12.2%) or MA (12.5%) (Table 1).

**Table 1.** Sample Characteristics by Medicare Plan Switching Patterns

Characteristic	Continuous		TM to MA (2861 [1.5%])	MA to TM (3534 [1.8%])	Other (623 [0.3%])	Total (196 536 [100.0%])	*P* value
	TM (128 095 [65.2%])	MA (61 423 [31.3%])					
Cancer site, No. (%)							
Breast	7176 (5.6)	3278 (5.3)	171 (6.0)	189 (5.3)	59 (9.5)	10873 (5.5)	<.001
Colorectal	19 126 (14.9)	8925 (14.5)	482 (16.8)	530 (15.0)	99 (15.9)	29 162 (14.8)	
NSCL	62 219 (48.6)	30 110 (49.0)	1281 (44.8)	1687 (47.7)	260 (41.7)	95 557 (48.6)	
Pancreas	14 152 (11.0)	6663 (10.8)	260 (9.1)	432 (12.2)	57 (9.1)	21 564 (11.0)	
Prostate	11 387 (8.9)	5890 (9.6)	355 (12.4)	317 (9.0)	74 (11.9)	18 023 (9.2)	
SCL	14 035 (11.0)	6557 (10.7)	312 (10.9)	379 (10.7)	74 (11.9)	21 357 (10.9)	
Age group at diagnosis, No. (%)							
66-74 y	62 730 (49.0)	29 951 (48.8)	1694 (59.2)	1900 (53.8)	370 (59.4)	96 645 (49.2)	<.001
75-84 y	48 965 (38.2)	24 513 (39.9)	921 (32.2)	1341 (37.9)	200 (32.1)	75 940 (38.6)	
≥85 y	16 400 (12.8)	6959 (11.3)	246 (8.6)	293 (8.3)	53 (8.5)	23 951 (12.2)	
Sex, No. (%)							
Male	68 775 (53.7)	32 401 (52.8)	1674 (58.5)	1866 (52.8)	343 (55.1)	105 059 (53.5)	<.001
Female	59 320 (46.3)	29 022 (47.2)	1187 (41.5)	1668 (47.2)	280 (44.9)	91 477 (46.5)	
Race and ethnicity, No. (%)^a^							
Hispanic	8175 (6.4)	6554 (10.7)	363 (12.7)	439 (12.4)	126 (20.2)	15 657 (8.0)	<.001
Non–Hispanic Black	11 476 (9.0)	8292 (13.5)	554 (19.4)	572 (16.2)	166 (26.6)	21 060 (10.7)	
Non–Hispanic White	102 549 (80.1)	43 336 (70.6)	1761 (61.6)	2185 (61.8)	266 (42.7)	150 097 (76.4)	
Other/unknown^b^	5895 (4.6)	3241 (5.3)	183 (6.4)	338 (9.6)	65 (10.5)	9722 (5.0)	
Metropolitan residency, No. (%)^c^							
Nonmetropolitan	19 508 (15.2)	5254 (8.6)	315 (11.0)	314 (8.9)	47 (7.5)	25 438 (12.9)	<.001
Metropolitan	108 587 (84.8)	56 169 (91.4)	2546 (89.0)	3220 (91.1)	576 (92.5)	171 098 (87.1)	
Marital status, No. (%)							
Married	42 033 (32.8)	20 171 (32.8)	916 (32.0)	828 (23.4)	149 (23.9)	64 097 (32.6)	<.001
Not married	35 470 (27.7)	16 633 (27.1)	837 (29.3)	862 (24.4)	169 (27.1)	53 971 (27.5)	
Unknown	50 592 (39.5)	24 619 (40.1)	1108 (38.7)	1844 (52.2)	305 (49.0)	78 468 (39.9)	
Yost quintile, No. (%)^d^							
First (lowest SES)	20 702 (16.2)	11 141 (18.1)	685 (23.9)	710 (20.1)	186 (29.9)	33 424 (17.0)	<.001
Second	21 967 (17.1)	10 332 (16.8)	506 (17.7)	613 (17.3)	119 (19.1)	33 537 (17.1)	
Third	23 690 (18.5)	11 872 (19.3)	554 (19.4)	660 (18.7)	110 (17.7)	36 886 (18.8)	
Fourth	27 353 (21.4)	13 645 (22.2)	559 (19.5)	730 (20.7)	94 (15.1)	42 381 (21.6)	
Fifth (highest SES)	33 631 (26.3)	14 029 (22.8)	538 (18.8)	786 (22.2)	>103 (>16.5)	49 089 (25.0)	
Unknown	752 (0.6)	404 (0.7)	19 (0.7)	35 (1.0)	<11 (<1.8)	1219 (0.6)	
Dual Medicare-Medicaid eligibility, No. (%)^e^							
Never dual	101 767 (79.4)	49 090 (79.9)	1975 (69.0)	2090 (59.1)	263 (42.2)	155 185 (79.0)	<.001
Gain dual	2615 (2.0)	1551 (2.5)	122 (4.3)	259 (7.3)	44 (7.1)	4591 (2.3)	
Lose dual	6785 (5.3)	2305 (3.8)	186 (6.5)	295 (8.3)	102 (16.4)	9673 (4.9)	
Always dual	15 646 (12.2)	7689 (12.5)	523 (18.3)	785 (22.2)	191 (30.7)	24 834 (12.6)	
Other	1282 (1.0)	788 (1.3)	55 (1.9)	105 (3.0)	23 (3.7)	2253 (1.1)	

Abbreviations: MA, Medicare Advantage; NSCL, non–small cell lung; SCL, small cell lung; SES, socioeconomic status; TM, traditional Medicare.

^a^ Race and ethnicity information was abstracted from medical records by cancer registrars at the time of diagnosis.

^b^ Other group includes American Indian/Alaska Native, Asian/Pacific Islander, and other unspecified. The unknown category has been reported with other to avoid reporting small numbers in accordance with the National Cancer Institute data use agreement.

^c^ Based on 2013 Rural Urban Continuum Code. Codes 1-3 were classified as metropolitan and codes 4-9 were classified as nonmetropolitan.

^d^ The Yost index is a composite SES score for census tracts based on median household income, median house value, median rent, percentage below 150% of the federal poverty line, Education Index, percentage working class, and percentage unemployed. Lower quintiles represent the lower SES groups.

^e^ With state buy-in (Medicaid) from cancer diagnosis to the end of follow-up.

### Hospice Utilization by Medicare Plan Switching Patterns

**Figure 1.**
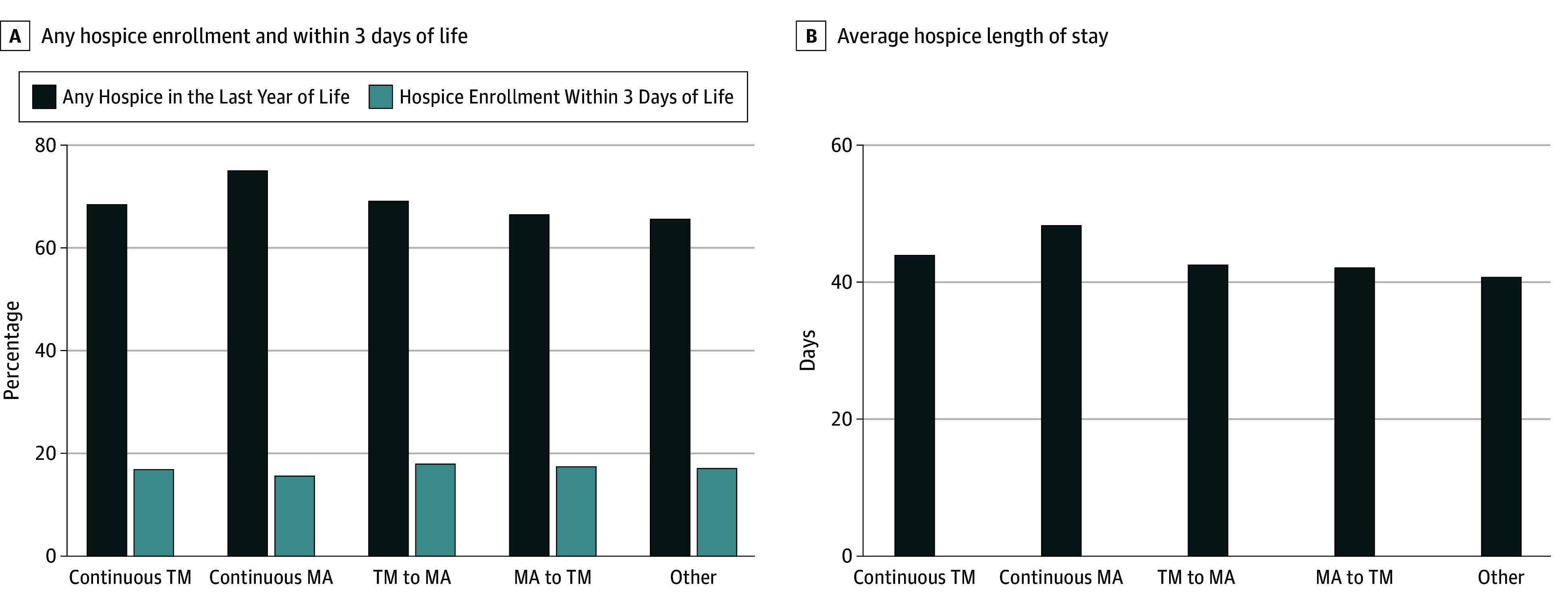
Bar Graphs Showing Hospice Utilization in the Last Year of Life MA indicates Medicare Advantage; TM, traditional Medicare.

Hospice enrollment in the last year of life was highest among beneficiaries with continuous MA (74.8%), followed by those who switched from TM to MA (69.0%), those with continuous TM (68.5%), and those who switched from MA to TM (66.4%; *P* < .001) (Figure 1). Such difference persisted in multivariable logistic regression, where beneficiaries with continuous MA had 6.93 percentage points (pp; 95% CI, 6.50-7.37 pp) higher likelihood of hospice enrollment than those with continuous TM coverage. Similarly, beneficiaries who switched from TM to MA and from MA to TM had 2.92 pp (95% CI, 1.30-4.54 pp) and 2.77 pp (95% CI, 1.32-4.22 pp) higher likelihood of hospice enrollment than those with continuous TM. In addition, beneficiaries diagnosed with pancreatic cancers had considerably higher hospice enrollment than those with breast cancers (11.80 pp; 95% CI, 10.72-12.88 pp). Compared with non-Hispanic White beneficiaries, non-Hispanic Black and Hispanic beneficiaries had −8.56 pp (95% CI, –9.30 to −7.82 pp) and −2.62 pp (95% CI, –3.42 to −1.83) lower likelihood of hospice enrollment (Table 2).

**Table 2.** Medicare Plan Switching Patterns and Patient Characteristics Associated With Hospice Enrollment^a^

Characteristic	Any hospice in the last year of life		Hospice in the last 3 d of life		Hospice length of stay	
	Adjusted percentage point difference (95% CI)	*P* value	Adjusted percentage point difference (95% CI)	*P* value	Adjusted differences in days (95% CI)	*P* value
Total No.	196 536		138 417		138 417	
Medicare plan switching						
Full TM	[Reference]	NA	[Reference]	NA	[Reference]	NA
TM to MA	2.92 (1.30 to 4.54)	<.001	0.04 (−1.60 to 1.69)	.96	0.28 (−0.02 to 0.58)	.06
MA to TM	2.77 (1.32 to 4.22)	<.001	0.44 (−1.10 to 1.99)	.57	−0.63 (−0.91 to −0.36)	<.001
Full MA	6.93 (6.50 to 7.37)	<.001	−1.39 (−1.83 to −0.96)	<.001	5.13 (5.05 to 5.22)	<.001
Other	2.65 (−0.73 to 6.03)	.13	−0.03 (−3.65 to 3.59)	.99	−3.41 (−4.02 to −2.80)	<.001
Cancer site						
Breast	[Reference]	NA	[Reference]	NA	[Reference]	NA
Colorectal	6.53 (5.47 to 7.58)	<.001	−5.28 (−6.36 to −4.20)	<.001	4.01 (3.84 to 4.19)	<.001
NSCL	3.04 (2.06 to 4.02)	<.001	−2.82 (−3.83 to −1.80)	<.001	0.88 (0.72 to 1.04)	<.001
Pancreas	11.80 (10.72 to 12.88)	<.001	−6.32 (−7.42 to −5.21)	<.001	−7.08 (−7.26 to −6.90)	<.001
Prostate	0.38 (−0.83 to 1.59)	.54	−3.00 (−4.24 to −1.76)	<.001	1.78 (1.57 to 1.98)	<.001
SCL	5.97 (4.87 to 7.08)	<.001	−2.45 (−3.59 to −1.31)	<.001	−8.21 (−8.39 to −8.04)	<.001
Age group at diagnosis						
66-74 y	[Reference]	NA	[Reference]	NA	[Reference]	NA
75-84 y	4.65 (4.23 to 5.08)	<.001	−2.92 (−3.35 to −2.49)	<.001	9.28 (9.21 to 9.36)	<.001
≥85 y	8.79 (8.18 to 9.40)	<.001	−6.11 (−6.68 to −5.53)	<.001	24.81 (24.68 to 24.94)	<.001
Sex						
Male	[Reference]	NA	[Reference]	NA	[Reference]	NA
Female	6.00 (5.56 to 6.44)	<.001	−3.00 (−3.43 to −2.57)	<.001	6.38 (6.30 to 6.45)	<.001
Race and ethnicity^a^						
Hispanic	−2.62 (−3.42 to −1.83)	<.001	−0.41 (−1.20 to 0.38)	.31	−1.70 (−1.84 to −1.56)	<.001
Non–Hispanic-Black	−8.56 (−9.30 to −7.82)	<.001	−0.07 (−0.79 to 0.65)	.84	−1.71 (−1.84 to −1.58)	<.001
Non–Hispanic-White	[Reference]	NA	[Reference]	NA	[Reference]	NA
Other^b^	−10.45 (−11.48 to −9.42)	<.001	0.84 (−0.25 to 1.92)	.13	−2.19 (−2.37 to −2.01)	<.001
Unknown	−3.43 (−10.41 to 3.55)	.34	−1.68 (−8.59 to 5.23)	.63	−0.62 (−1.87 to 0.62)	.33
Metropolitan residency^c^						
Nonmetropolitan	[Reference]	NA	[Reference]	NA	[Reference]	NA
Metropolitan	0.70 (−0.01 to 1.41)	.06	0.14 (−0.55 to 0.82)	.70	1.13 (1.01 to 1.25)	<.001
Marital status						
Married	[Reference]	NA	[Reference]	NA	[Reference]	NA
Not married	0.70 (0.16 to 1.24)	.01	−1.52 (−2.05 to −0.99)	<.001	4.85 (4.75 to 4.94)	<.001
Unknown	−0.23 (−1.45 to 0.99)	.71	−1.38 (−2.59 to −0.16)	.03	5.26 (5.05 to 5.47)	<.001
Yost quintile^d^						
First (lowest SES)	[Reference]	NA	[Reference]	NA	[Reference]	NA
Second	0.34 (−0.37 to 1.04)	.35	0.32 (−0.37 to 1.00)	.36	−1.87 (−1.99 to −1.74)	<.001
Third	1.25 (0.54 to 1.96)	.001	0.47 (−0.22 to 1.16)	.18	−2.42 (−2.55 to −2.29)	<.001
Fourth	2.30 (1.58 to 3.01)	<.001	0.51 (−0.19 to 1.21)	.15	−2.54 (−2.67 to −2.41)	<.001
Fifth (highest SES)	1.83 (1.10 to 2.55)	<.001	1.17 (0.45 to 1.89)	.001	−4.42 (−4.55 to −4.29)	<.001
Unknown	0.37 (−2.10 to 2.85)	.77	0.97 (−1.68 to 3.61)	.48	0.87 (0.36 to 1.38)	.001
Dual Medicare-Medicaid eligibility^e^						
Never dual	[Reference]	NA	[Reference]	NA	[Reference]	NA
Gain dual	1.69 (0.42 to 2.96)	.01	−3.92 (−5.08 to −2.76)	<.001	14.36 (14.09 to 14.62)	<.001
Lose dual	−7.14 (−8.20 to −6.08)	<.001	0.30 (−0.84 to 1.43)	.61	7.65 (7.45 to 7.85)	<.001
Always dual	−3.09 (−3.75 to −2.44)	<.001	−2.03 (−2.66 to −1.40)	<.001	8.75 (8.62 to 8.88)	<.001
Other	−1.08 (−2.93 to 0.78)	.26	−2.14 (−3.96 to −0.32)	.02	11.73 (11.36 to 12.11)	<.001
MA penetration rate	4.15 (2.20 to 6.10)	<.001	2.42 (0.43 to 4.42)	.02	−12.33 (−12.69 to −11.98)	<.001

Abbreviations: NA, not applicable; other acronym expansions in Table 1 footnote.

^a^ Footnote a in Table 1 gives race and ethnicity information.

^b^ Footnote b in Table 1 gives other group information.

^c^ Footnote c in Table 1 gives Rural Urban Continuum Code information.

^d^ Footnote d in Table 1 gives Yost index information.

^e^ Footnote e in Table 1 gives eligibility information.

Among hospice users, enrollment within 3 days of death was lower among continuous MA enrollees (15.5%) compared with other groups: 17.1% with continuous TM, 18.0% among those who switched from TM to MA, and 17.6% among those who switched from MA to TM. In adjusted analysis, hospice enrollment within 3 days of death was lower among beneficiaries with continuous MA (−1.39 pp; 95% CI, –1.83 to −0.96 pp) compared with beneficiaries with continuous TM, but similar among those who switched from TM to MA (0.04 pp; 95% CI, –1.60 to 1.69 pp) and those who switched from MA to TM (0.44 pp; 95% CI, –1.10 to 1.99 pp).

Among hospice users, the average total hospice length of stay was longer among beneficiaries with continuous MA (48.3 days) than those with continuous TM (43.8 days), those who switched from TM to MA (42.7 days), and those who switched from MA to TM (42.1 days). Adjusted regression analysis showed persistent positive differences in hospice length of stay (5.13 days; 95% CI, 5.05-5.22 days) for beneficiaries with continuous MA than for those with continuous TM.

### Place of Hospice Care by Medicare Plan Switching Patterns

**Figure 2.**
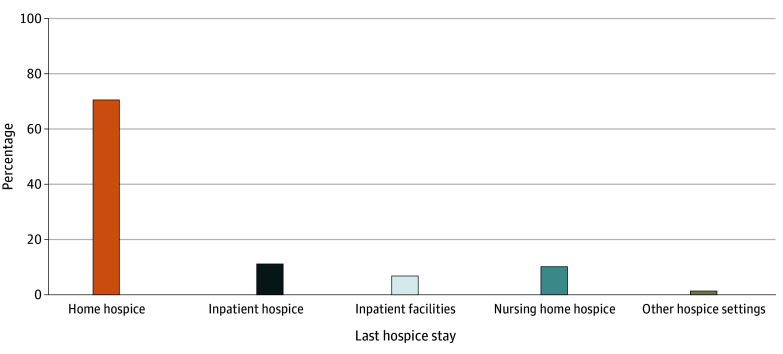
Bar Graph Showing the Distribution of Place of Last Hospice Stay

The last hospice service was received at home by 70.4% of hospice enrollees, followed by 10.8% at hospice facilities, 9.6% at nursing homes, and 7.6% at inpatient facilities (Figure 2). Compared with continuous TM, continuous MA beneficiaries had 1.93 pp (95% CI, 1.40-2.45 pp) higher likelihood of receiving their last hospice service at home. In addition, beneficiaries who were older (≥85 years vs 66-74 years: 2.43 pp; 95% CI, 1.70-3.16 pp), female (1.88 pp; 95% CI, 1.37-2.39 pp), and Hispanic (vs non-Hispanic White: 4.89 pp; 95% CI, 3.96-5.81 pp) were more likely to receive home hospice, while non-Hispanic Black beneficiaries (−1.95 pp; 95% CI, –2.80 to −1.10 pp) and those with newly gained dual coverage (−31.98 pp; 95% CI, –33.69 to −30.27 pp) were less likely to receive home hospice (Table 3).

**Table 3.** Medicare Plan Switching Patterns and Patient Characteristics Associated With Place of Last Hospice Stay^a^

Characteristic	Home hospice		Nursing home hospice	
	Adjusted probability difference (95% CI)	*P* value	Adjusted probability difference (95% CI)	*P* value
Total No.	138 417	NA	138 417	NA
Medicare plan switching				
Full TM	[Reference]	NA	[Reference]	NA
TM to MA	1.75 (−0.16 to 3.65)	.07	−0.77 (−1.93 to 0.38)	.19
MA to TM	−3.26 (−5.08 to −1.45)	<.001	2.45 (1.26 to 3.63)	<.001
Full MA	1.93 (1.40 to 2.45)	<.001	−1.83 (−2.16 to −1.51)	<.001
Other	1.93 (−2.14 to 6.01)	.35	0.21 (−2.15 to 2.58)	.86
Cancer site				
Breast	[Reference]	NA	[Reference]	NA
Colorectal	3.97 (2.76 to 5.18)	<.001	−1.91 (−2.69 to −1.13)	<.001
NSCL	3.57 (2.46 to 4.69)	<.001	−2.60 (−3.32 to −1.88)	<.001
Pancreas	7.29 (6.04 to 8.54)	<.001	−4.98 (−5.78 to −4.19)	<.001
Prostate	0.92 (−0.51 to 2.34)	.21	0.27 (−0.68 to 1.22)	.58
SCL	5.62 (4.36 to 6.87)	<.001	−3.41 (−4.21 to −2.60)	<.001
Age group at diagnosis				
66-74 y	[Reference]	NA	[Reference]	NA
75-84 y	0.99 (0.49 to 1.50)	<.001	1.60 (1.29 to 1.92)	<.001
≥85 y	2.43 (1.70 to 3.16)	<.001	3.63 (3.14 to 4.13)	<.001
Sex				
Male	[Reference]	NA	[Reference]	NA
Female	1.88 (1.37 to 2.39)	<.001	−0.62 (−0.95 to −0.29)	<.001
Race and ethnicity^a^				
Hispanic	4.89 (3.96 to 5.81)	<.001	−4.55 (−4.98 to −4.13)	<.001
Non–Hispanic-Black	−1.95 (−2.80 to −1.10)	<.001	−0.73 (−1.23 to −0.23)	.004
Non–Hispanic-White	[Reference]	NA	[Reference]	NA
Other^b^	0.03 (−1.32 to 1.37)	.97	−1.30 (−1.99 to −0.62)	<.001
Unknown	−3.44 (−13.06 to 6.18)	.48	−5.15 (−8.70 to −1.59)	.005
Metropolitan residency^c^				
Nonmetropolitan	[Reference]	NA	[Reference]	NA
Metropolitan	−3.72 (−4.49 to −2.95)	<.001	−0.25 (−0.76 to 0.25)	.33
Marital status				
Married	[Reference]	NA	[Reference]	NA
Not married	−5.83 (−6.49 to −5.17)	<.001	4.08 (3.67 to 4.48)	<.001
Unknown	−1.48 (−2.93 to −0.02)	.05	3.11 (2.27 to 3.94)	<.001
Yost quintile^d^				
First (lowest SES)	[Reference]	NA	[Reference]	NA
Second	0.31 (−0.49 to 1.12)	.45	0.49 (−0.01 to 0.98)	.06
Third	−0.62 (−1.43 to 0.19)	.14	0.20 (−0.31 to 0.70)	.45
Fourth	−0.48 (−1.31 to 0.35)	.26	−0.03 (−0.55 to 0.48)	.90
Fifth (highest SES)	0.18 (−0.67 to 1.03)	.69	−0.77 (−1.29 to −0.24)	.004
Unknown	−5.63 (−8.81 to −2.45)	.001	1.36 (−0.56 to 3.29)	.16
Dual Medicare Medicaid eligibility^e^				
Never dual	[Reference]	NA	[Reference]	NA
Gain dual	−31.98 (−33.69 to −30.27)	<.001	33.76 (32.10 to 35.43)	<.001
Lose dual	−18.72 (−20.25 to −17.19)	<.001	18.60 (17.26 to 19.94)	<.001
Always dual	−15.55 (−16.40 to −14.70)	<.001	16.54 (15.81 to 17.27)	<.001
Other	−21.12 (−23.60 to −18.64)	<.001	23.23 (20.98 to 25.47)	<.001
MA penetration rate	−8.64 (−11.06 to −6.22)	<.001	0.59 (−0.94 to 2.12)	.45

Abbreviations: MA, Medicare Advantage; NA, not applicable; NSCL, non–small cell lung; SCL, small cell lung; Ref, reference; SES, socioeconomic status; TM, traditional Medicare.

^a^ Race and ethnicity information was abstracted from medical records by cancer registrars at the time of diagnosis.

^b^ Other group includes American Indian/Alaska Native, Asian/Pacific Islander, and other unspecified.

^c^ Based on 2013 Rural Urban Continuum Code. Codes 1-3 were classified as metropolitan and codes 4-9 were classified as nonmetropolitan.

^d^ The Yost index is a composite SES score for census tracts based on median household income, median house value, median rent, percentage below 150% of federal poverty line, Education Index, percentage working class, and percentage unemployed. Lower quintiles represent the lower SES groups.

^e^ With state buy-in (Medicaid) from cancer diagnosis to the end of follow-up.

In contrast, those who switched from MA to TM had 2.45 pp (95% CI, 1.26-3.63 pp) higher likelihood and those with continuous MA had −1.83 pp (95% CI, –2.16 to −1.51 pp) lower likelihood of receiving last hospice service at nursing homes than beneficiaries with continuous TM, after adjusting for patient characteristics. Racial and ethnic minority groups, including Hispanic (−4.55 pp; 95% CI, –4.98 to −4.13 pp), non-Hispanic Black (−0.73 pp; 95% CI, –1.23 to −0.23 pp), and non-Hispanic other (−1.30 pp; 95% CI, –1.99 to −0.62 pp) beneficiaries were less likely to receive hospice at nursing homes. Dual Medicare and Medicaid coverage had highest adjusted probability difference values with nursing home use, where those gaining dual coverage was associated with 33.76 pp (95% CI, 32.10-35.43 pp) higher likelihood of hospice use at a nursing home compared with those without dual coverage (Table 3).

### Sensitivity Analyses

Sensitivity analyses among those who survived 12 months showed a lower percentage of beneficiaries with pancreatic cancer, NSCLC, and SCLC than the primary sample (eTable 3 in Supplement 1), but consistent statistical significance and direction of the adjusted probability differences between plan switching patterns and hospice utilization and place of hospice care (eTable 4 in Supplement 1). When stratified by any dual Medicare and Medicaid coverage, those with continuous MA coverage consistently had a higher likelihood of hospice utilization than those with continuous TM coverage, although the magnitude was higher among dual than nondual beneficiaries (9.15 pp [95% CI, 8.10-10.20 pp] and 6.32 pp [95% CI, 5.81-6.84 pp]) (eTables 5 and 6 in Supplement 1). Similar patterns were observed for hospice use at home and in a nursing home: the increased likelihood of home hospice among beneficiaries with continuous MA (vs continuous TM) was higher among dual (2.71 pp; 95% CI, 1.36-4.06 pp) than nondual (1.63 pp; 95% CI, 1.05-2.21 pp) beneficiaries; and the increased likelihood of hospice at nursing homes among MA-to-TM beneficiaries was higher among dual (6.01 pp; 95% CI, 2.80-9.21 pp) than nondual (1.27 pp; 95% CI, 0.16-2.37 pp) beneficiaries (eTables 5 and 6 in Supplement 1). Regression results with and without adjusting for NCI comorbidity index were similar and consistent with the primary analysis (eTable 7 in Supplement 1). Among the subcohort with SNF data available, we found a higher rate of MA-to-TM switching (3.0%) among those with an SNF stay after cancer diagnosis than those without (1.5%; eTable 8 in Supplement 1); and the adjusted probability differences in nursing home hospice use between MA to TM and continuous TM became smaller (2.38 pp; 95%- CI, 0.66-4.10 pp) after adjusting for SNF stay than without adjustments (3.54 pp; 95% CI, 1.62-5.45). (eTable 9 in Supplement 1).

## Discussion

In this large, population-based cohort of Medicare beneficiaries diagnosed with distant-stage cancers, plan switching between MA and TM in the last year of life was relatively infrequent. However, we found distinct patterns of hospice utilization and place of last hospice service across plan switching patterns.

Our findings extend prior research showing that MA enrollees are more likely to use hospice than TM enrollees.^11,32,33^ Importantly, we found longer hospice stays and lower rates of late hospice enrollment among continuous MA enrollees, suggesting that higher overall hospice use among MA beneficiaries was driven by earlier transition into hospice.^12^ This aligns with the hypothesis that MA plans proactively encourage hospice enrollment, whether through financial incentives (eg, utilization management, provider networks) to avoid costly hospitalizations, care management approaches that facilitate EOL transitions, or better integration of supportive care services.^12,34^ Our study cannot directly test these mechanisms; further investigation to identify which aspects of plan benefit design may support timely, preference-concordant EOL care across both MA and TM is warranted.

We also examined how transitions between programs are associated with the setting of EOL care. Continuous MA enrollees were more likely to receive hospice care at home, consistent with prior evidence that MA decedents more often enter hospice from community settings.^12^ While patient preferences for place of EOL care are highly contingent on clinical and social context, home remains the most commonly preferred place of EOL care for patients and families, particularly when symptom burden is manageable and informal caregiving support is available.^35^ In contrast, those who switched from MA to TM were more likely to receive hospice care in nursing homes. Although MA is not responsible for administering and managing hospice care, several MA features may explain this pattern. First, MA plans maintain narrower SNF networks and stricter prior authorization and length-of-stay for SNF stay limits than TM.^36^ Patients with advanced cancer who require postacute or rehabilitative care facing access barriers under MA may switch to TM for broader SNF coverage. The SNF facility may become the care setting where patients ultimately receive their hospice services. Our sensitivity analyses confirmed this potential mechanism (ie, mediation): (1) the MA-to-TM switching rate was higher among those with an SNF stay after cancer diagnosis than those without and (2) the adjusted probability differences in nursing home hospice use between MA to TM and continuous TM became smaller after adjusting for SNF stay.

Another potential explanation for the higher use of nursing home hospice among those who switched from MA to TM relates to the integration of Medicare and Medicaid in financing of nursing home care. Although MA plans manage Medicare-covered services, custodial long-term nursing home care is covered by Medicaid.^37,38^ Many dual-eligible beneficiaries in MA must still navigate 2 separate programs, unless they are in a fully integrated plan such as a Fully Integrated Dual-Eligible Special Needs Plan,^29^ which could create administrative complexity, limited overlaps between MA networks and Medicaid-covered nursing homes, and fewer facility choices.^36,39,40^ For patients with advanced cancer and substantial nursing home needs, these barriers may prompt switching to TM, which simplifies coordination with Medicaid-covered long-term care. Once in nursing homes under TM, transitions into hospice at the same facility may follow more directly. Consistent with this, prior research shows that dual Medicare-Medicaid enrollees are more likely to disenroll from MA than non-dual enrollees.^41^ We similarly found switching was more common among dual enrollees, a group characterized by both greater clinical complexity and higher likelihood of needing intensive nursing care.^29,42,43,44^ Moreover, the associations between MA-to-TM switching and nursing home hospice use were larger among dually covered than non–dually covered beneficiaries. These findings suggest that structural barriers to long-term care access in MA, combined with Medicaid’s coverage of room and board for nursing home care, may drive plan switching and subsequent receipt of hospice in nursing homes.

These results highlight important policy implications related to structural constraints from benefit design and plan switching rules that may influence where hospice care is ultimately received. Although home remains the most preferred place of death, population aging, multimorbidity, and declines in family caregiving capacity are likely to increase demand for nursing home–based hospice,^45,46^ particularly among individuals residing in long-term care facilities who may even view the nursing home as their “home” at the EOL.^35,47^ In this context, higher nursing home hospice use among those who switched from MA to TM may reflect structural mismatches between care needs, benefit design, and beneficiaries’ capacity to navigate coverage options. For example, dual Medicare-Medicaid enrollees benefit from Special Enrollment Periods that allow plan changes once per quarter and, beginning in January 2025, will permit monthly switches.^48^ In contrast, non–dually covered beneficiaries face a narrower annual enrollment period.^13^ Their choices are further constrained by financial barriers to obtaining supplemental coverage under TM. Because TM lacks an annual out-of-pocket maximum, many beneficiaries rely on Medigap policies to offset cost-sharing.^49^ Yet, in most states, Medigap insurance providers may deny coverage or charge prohibitively high premiums based on preexisting conditions.^50,51^ For patients newly diagnosed with cancer, these barriers can effectively lock them into MA plans even when they are facing narrow networks or other unmet needs.

### Limitations

Our study has limitations. First, causal relationships between plan switching and hospice utilization or place of hospice cannot be inferred from this observational study. Instead, those who switch from MA to TM may represent distinct subgroups with underlying nursing care needs. Second, our outcome measures such as late hospice enrollment and total length of hospice stay may not capture all dimensions of hospice quality. We also could not capture several unmeasured confounders, such as patients’ or family members’ underlying preference for place of hospice care (eg, home, nursing home), which may be influenced by programmatic constraints, caregiver support, and other factors. Last, findings were restricted to older Medicare beneficiaries diagnosed with 6 common solid tumors within SEER regions and may not be generalizable to other patient populations.

## Conclusions

This retrospective cohort study of Medicare decedents diagnosed with distant-stage cancers in 2010 to 2019 found that hospice utilization patterns at the EOL varied substantially by Medicare plan type and plan switching behavior. Continuous MA enrollment was associated with higher hospice use and more frequently home hospice, while MA-to-TM switching was associated with greater nursing home hospice use, particularly among dually covered beneficiaries. These findings suggest that insurance program structures and enrollment policies influence where and how patients receive hospice care at the EOL. Future research to understand potential care coordination gaps, access to patient-centered hospice care settings, and barriers to plan switching for medically vulnerable beneficiaries is critical to identify targeted intervention to promote equitable and patient-centered EOL care.

## Notes

Supplement 1. **eFigure 1.** CONSORT Chart for creation of patient cohort **eFigure 2.** Examples of Medicare Plan Switching Patterns **eTable 1.** Cancer Identification **eTable 2.** Place Specific Healthcare Common Procedure Coding System for Hospice Services **eTable 3.** Sample Characteristics of Medicare Beneficiaries Who Survived At Least 12 Months **eTable 4.** Medicare Plan Switching and Patient Characteristics Associated with Hospice Enrollment and Place of Last Hospice Stay Among Beneficiaries Survived At Least 12 Months **eTable 5.** Medicare Plan Switching and Patient Characteristics Associated with Hospice Enrollment and Place of Last Hospice Stay Among Beneficiaries With Dual Medicaid and Medicare Enrollment **eTable 6.** Medicare Plan Switching and Patient Characteristics Associated with Hospice Enrollment and Place of Last Hospice Stay Among Beneficiaries Without Dual Medicaid and Medicare Enrollment **eTable 7.** Association Between Medicare Plan Switching and Hospice Enrollment and Place of Last Hospice Stay With and Without Adjusting for NCI Comorbidity Index **eTable 8.** Medicare Plan Switching Stratified by Skilled Nursing Facility Stay Post Cancer Diagnosis **eTable 9.** Medicare Plan Switching and Patient Characteristics Associated with Nursing Home Hospice Enrollment With and Without Adjusting for Skilled Nursing Facility Stay Post Cancer Diagnosis

Supplement 2.
Data Sharing Statement
