# Supplementary material for: Medicare Plan Switching and Hospice Care Among Decedents With Advanced Cancer
Source: JAMA Netw Open. 2026 Mar 24;9(3):e260755. doi: 10.1001/jamanetworkopen.2026.0755 (PMC13014202; doi:10.1001/jamanetworkopen.2026.0755)
Supplement: Supplement 1. — eFigure 1. CONSORT Chart for creation of patient cohort eFigure 2. Examples of Medicare Plan Switching Patterns eTable 1. Cancer Identification eTable 2. Place Specific Healthcare Common Procedure Coding System for Hospice Services eTable 3. Sample Characteristics of Medicare Beneficiaries Who Survived At Least 12 Months eTable 4. Medicare Plan Switching and Patient Characteristics Associated with Hospice Enrollment and Place of Last Hospice Stay Among Beneficiaries Survived At Least 12 Months eTable 5. Medicare Plan Switching and Patient Characteristics Associated with Hospice Enrollment and Place of Last Hospice Stay Among Beneficiaries With Dual Medicaid and Medicare Enrollment eTable 6. Medicare Plan Switching and Patient Characteristics Associated with Hospice Enrollment and Place of Last Hospice Stay Among Beneficiaries Without Dual Medicaid and Medicare Enrollment eTable 7. Association Between Medicare Plan Switching and Hospice Enrollment and Place of Last Hospice Stay With and Without Adjusting for NCI Comorbidity Index eTable 8. Medicare Plan Switching Stratified by Skilled Nursing Facility Stay Post Cancer Diagnosis eTable 9. Medicare Plan Switching and Patient Characteristics Associated with Nursing Home Hospice Enrollment With and Without Adjusting for Skilled Nursing Facility Stay Post Cancer Diagnosis [file jamanetwopen-e260755-s001.pdf]

Supplemental Online Content

Hu X, Jiang C, Kwon Y, et al. Medicare plan switching and hospice care among decedents with advanced cancer. *JAMA Netw Open*. 2026;9(3):e260755. doi:10.1001/jamanetworkopen.2026.0755

eFigure 1. CONSORT Chart for Creation of Patient Cohort

eFigure 2. Examples of Medicare Plan Switching Patterns

eTable 1. Cancer Identification

eTable 2. Place Specific Healthcare Common Procedure Coding System for Hospice Services

eTable 3. Sample Characteristics of Medicare Beneficiaries Who Survived At Least 12 Months

eTable 4. Medicare Plan Switching and Patient Characteristics Associated with Hospice Enrollment and Place of Last Hospice Stay Among Beneficiaries Survived At Least 12 Months

eTable 5. Medicare Plan Switching and Patient Characteristics Associated with Hospice Enrollment and Place of Last Hospice Stay Among Beneficiaries With Dual Medicaid and Medicare Enrollment

eTable 6. Medicare Plan Switching and Patient Characteristics Associated with Hospice Enrollment and Place of Last Hospice Stay Among Beneficiaries Without Dual Medicaid and Medicare Enrollment

eTable 7. Association Between Medicare Plan Switching and Hospice Enrollment and Place of Last Hospice Stay With and Without Adjusting for NCI Comorbidity Index

eTable 8. Medicare Plan Switching Stratified by Skilled Nursing Facility Stay Post Cancer Diagnosis

eTable 9. Medicare Plan Switching and Patient Characteristics Associated with Nursing Home Hospice Enrollment With and Without Adjusting for Skilled Nursing Facility Stay Post Cancer Diagnosis

This supplemental material has been provided by the authors to give readers additional information about their work.

© 2026 Hu X et al. *JAMA Network Open*.

eFigure 1. CONSORT Chart for Creation of Patient Cohort

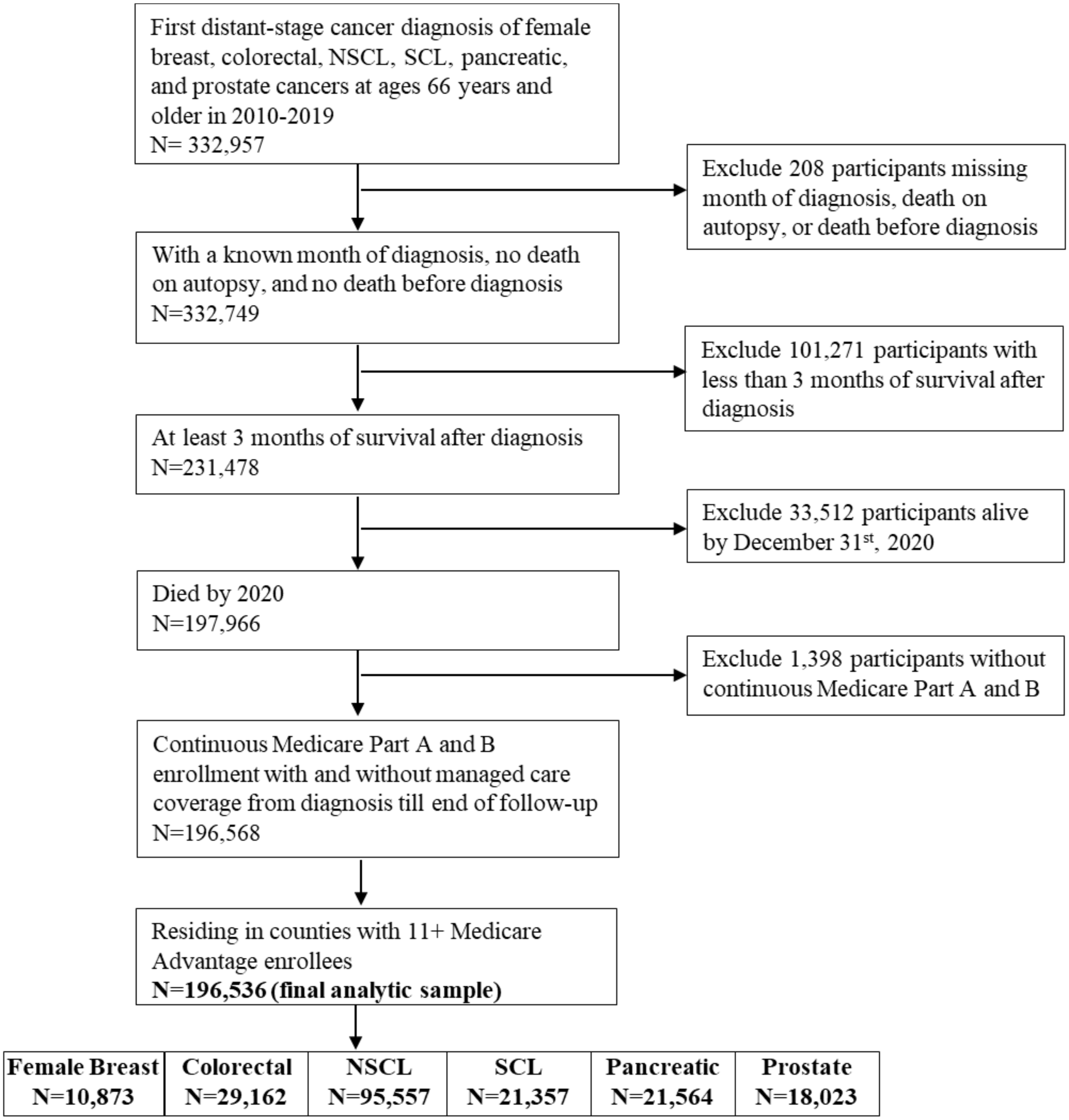

* *Notes*: NSCL = Non-Small Cell Lung; SCL = Small Cell Lung.

© 2026 Hu X et al. *JAMA Network Open*.

eFigure 2. Examples of Medicare Plan Switching Patterns

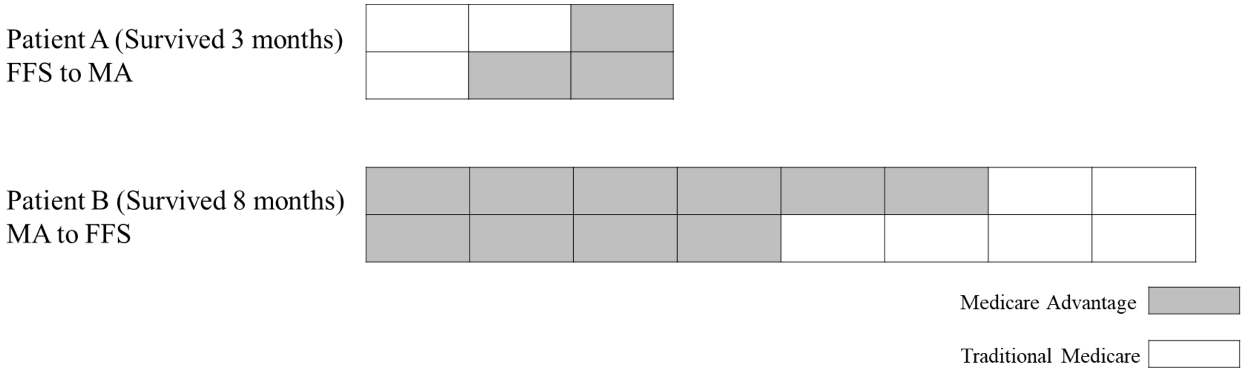

© 2026 Hu X et al. *JAMA Network Open*.

eTable 1. Cancer Identification

Cancer Type	Cancer Site	Histology
Female Breast	ICD-O-3: C500-C509; SEER Site-recode: 26000	8140,8141,8143,8147,8500,8501,8502,8503,8504, 8507,8508,8509,8520,8521,8522,8523,8524,8525, 8570,8571,8572,8573,8574,8575
Colorectal	ICD-O-3: C180-C189, C199, C209; SEER Site-recode: 21041-21049, 21051-21052, 21060	8140, 8210, 8211, 8220, 8221, 8260, 8261, 8262, 8263, 8470, 8480, 8481, 8490
NSCLC	ICD-O-3: C34.0-C34.3, C34.8, C34.9, C33.9; SEER Site-recode: 22030	8075,8076,8078,8140,8141,8143,8147, 8250,8251, 8252,8253,8254,8255,8260,8310,8430,8480,8481, 8560,8570,8571,8572,8573,8574
SCLC	ICD-O-3: C34.0-C34.3, C34.8, C34.9, C33.9; SEER Site-recode: 22030	8041, 8042, 8043, 8044, 8045
Pancreas	ICD-O-3: C250-C259; SEER Site-recode: 21100	8140, 8141,8143,8211,8500,8501,8503,8504,8508
Prostate	ICD-O-3: C619; SEER Site-recode: 28010	8140

Notes: Distant stage at diagnosis identified using SEER Combined Summary Stage. NSCLC = Non-Small Cell Lung Cancer; SCLC=Small Cell Lung Cancer.

© 2026 Hu X et al. *JAMA Network Open*.

eTable 2. Place Specific Healthcare Common Procedure Coding System and for Hospice Services

Settings	HCPCS
Home	Q5001 – Home<br>Q5002 – Assisted living facility
Nursing Home	Q5003 – Long term care or non-skilled nursing facility<br>Q5004 – Skilled nursing facility
Inpatient Facilities	Q5005 – Inpatient hospital
Inpatient Hospice	Q5006 – Inpatient hospice facility
Other	Q5007 – Long term care hospital<br>Q5008 – Inpatient psychiatric facility<br>Q5009 – Place not otherwise specified<br>Q5010 – Hospice residential facility

© 2026 Hu X et al. *JAMA Network Open*.

eTable 3. Sample Characteristics of Medicare Beneficiaries Who Survived At Least 12 Months

	Continuous TM	Continuous MA	TM to MA	MA to TM	Other	Total	P-value
**Total N, Row %**	60374 (65.3)	28406 (30.7)	1630 (1.8)	1616 (1.8)	410 (0.4)	92436 (100.0)	
**Cancer Site, N (Col %)**							<.0001
**Breast**	4934 (8.2)	2199 (7.7)	132 (8.1)	127 (7.9)	51 (12.4)	7443 (8.1)	
**Colorectal**	10752 (17.8)	4986 (17.6)	308 (18.9)	291 (18.0)	70 (17.1)	16407 (17.7)	
**NSCL**	26345 (43.6)	12630 (44.5)	675 (41.4)	688 (42.6)	166 (40.5)	40504 (43.8)	
**Pancreas**	4405 (7.3)	1787 (6.3)	94 (5.8)	137 (8.5)	23 (5.6)	6446 (7.0)	
**Prostate**	8631 (14.3)	4436 (15.6)	276 (16.9)	227 (14.0)	66 (16.1)	13636 (14.8)	
**SCL**	5307 (8.8)	2368 (8.3)	145 (8.9)	146 (9.0)	34 (8.3)	8000 (8.7)	
**Age Group At Diagnosis, N (Col %)**							<.0001
**66-74 y**	31382 (52.0)	14720 (51.8)	961 (59.0)	907 (56.1)	256 (62.4)	48226 (52.2)	
**75-84 y**	22288 (36.9)	10826 (38.1)	536 (32.9)	578 (35.8)	121 (29.5)	34349 (37.2)	
**≥85 y**	6704 (11.1)	2860 (10.1)	133 (8.2)	131 (8.1)	33 (8.0)	9861 (10.7)	
**Sex, N (Col %)**							<.0001
**Male**	32435 (53.7)	15259 (53.7)	949 (58.2)	876 (54.2)	229 (55.9)	49748 (53.8)	
**Female**	27939 (46.3)	13147 (46.3)	681 (41.8)	740 (45.8)	181 (44.1)	42688 (46.2)	
**Race and Ethnicity, N (Col %)**							<.0001
**Hispanic**	3928 (6.5)	3090 (10.9)	187 (11.5)	185 (11.4)	88 (21.5)	7478 (8.1)	
**Non-Hispanic Black**	5242 (8.7)	3994 (14.1)	314 (19.3)	268 (16.6)	115 (28.0)	9933 (10.7)	
**Non-Hispanic White**	48120 (79.7)	19618 (69.1)	1037 (63.6)	1029 (63.7)	169 (41.2)	69973 (75.7)	
**Other/Unknown** [a]	3084 (5.1)	1704 (6.0)	92 (5.7)	134 (8.3)	38 (9.2)	5052 (5.5)	
**Metropolitan Residency** [b]**, N (Col %)**							<.0001
**Non-metropolitan**	8821 (14.6)	2409 (8.5)	182 (11.2)	159 (9.8)	35 (8.5)	11606 (12.6)	
**Metropolitan**	51553 (85.4)	25997 (91.5)	1448 (88.8)	1457 (90.2)	375 (91.5)	80830 (87.4)	
**Marital Status, N (Col %)**							<.0001
**Married**	20583 (34.1)	9778 (34.4)	534 (32.8)	389 (24.1)	94 (22.9)	31378 (33.9)	
**Not married**	15730 (26.1)	7370 (25.9)	476 (29.2)	381 (23.6)	113 (27.6)	24070 (26.0)	
**Unknown**	24061 (39.9)	11258 (39.6)	620 (38.0)	846 (52.4)	203 (49.5)	36988 (40.0)	
**Yost Quintile** [c]**, N (Col %)**							<.0001
**First (Lowest SES)**	9135 (15.1)	4950 (17.4)	371 (22.8)	320 (19.8)	126 (30.7)	14902 (16.1)	
**Second**	10003 (16.6)	4654 (16.4)	288 (17.7)	286 (17.7)	79 (19.3)	15310 (16.6)	
**Third**	10985 (18.2)	5459 (19.2)	325 (19.9)	304 (18.8)	68 (16.6)	17141 (18.5)	
**Fourth**	13008 (21.5)	6363 (22.4)	300 (18.4)	333 (20.6)	65 (15.9)	20069 (21.7)	
**Fifth (Lowest SES)/Unknown**	17243 (28.6)	6980 (24.6)	346 (21.2)	373 (23.1)	72 (17.6)	25014 (27.1)	
**Dual Medicare Medicaid Eligibility** [d]**, N (Col %)**							<.0001
**Never Dual**	48227 (79.9)	22570 (79.5)	1112 (68.2)	983 (60.8)	171 (41.7)	73063 (79.0)	
**Gain Dual**	1187 (2.0)	785 (2.8)	72 (4.4)	122 (7.5)	30 (7.3)	2196 (2.4)	

© 2026 Hu X et al. *JAMA Network Open*.

**Lose Dual**	3029 (5.0)	1036 (3.6)	105 (6.4)	96 (5.9)	69 (16.8)	4335 (4.7)
**Always Dual**	7240 (12.0)	3592 (12.6)	308 (18.9)	360 (22.3)	125 (30.5)	11625 (12.6)
**Other**	691 (1.1)	423 (1.5)	33 (2.0)	55 (3.4)	15 (3.7)	1217 (1.3)

* *Notes*: MA=Medicare Advantage; NSCL = Non-Small Cell Lung; SCL = Small Cell Lung; SES = Socioeconomic Status; TM=Traditional Medicare.

[a] Other group includes American Indian/Alaska Native, Asian/Pacific Islander, and other unspecified.

[b] Based on 2013 Rural Urban Continuum Code. Codes 1-3 were classified as metropolitan, and Codes 4-9 were classified as Non-metropolitan.

[c] The Yost index is a composite SES scores for census tracts based on Median household income, Median house value, Median rent, Percent below 150% of federal poverty line, Education Index, Percent working class, and Percent unemployed. Lower quintiles represent the lower SES groups. The unknown category has been reported with the Fifth quintile to avoid reporting small numbers in accordance with the National Cancer Institute data use agreement.

[d] With state buy-in (Medicaid) from cancer diagnosis to 12 months after diagnosis.

© 2026 Hu X et al. *JAMA Network Open*.

eTable 4. Medicare Plan Switching and Patient Characteristics Associated with Hospice Enrollment and Place of Last Hospice Stay Among Beneficiaries Survived At Least 12 Months

	**Any Hospice In the Last Year of Life**		**Home Hospice In the Last Year of Life**		**Nursing Home Hospice In the Last Year of Life**	
	**Adjusted Probability Difference (95%CI)**	**P-value**	**Adjusted Probability Difference (95%CI)**	**P-value**	**Adjusted Probability Difference (95%CI)**	**P-value**
**Total N**	92436		65448		65448	
Medicare Plan Switching						
Full TM	Ref		Ref		Ref	
TM to MA	2.19 (0.02 to 4.35)	0.048	1.64 (-0.82 to 4.10)	0.191	-0.52 (-2.02 to 0.98)	0.494
MA to TM	2.62 (0.48 to 4.76)	0.017	-3.81 (-6.44 to -1.18)	0.005	2.28 (0.58 to 3.97)	0.009
Full MA	5.85 (5.20 to 6.49)	<.0001	2.18 (1.42 to 2.93)	<.0001	-1.79 (-2.25 to -1.32)	<.0001
Other	1.06 (-3.16 to 5.28)	0.623	2.10 (-2.83 to 7.03)	0.404	-0.13 (-2.95 to 2.69)	0.928
Cancer Site						
Breast	Ref		Ref		Ref	
Colorectal	7.51 (6.19 to 8.83)	<.0001	4.88 (3.38 to 6.38)	<.0001	-2.54 (-3.50 to -1.58)	<.0001
NSCL	2.27 (1.05 to 3.49)	<.0001	3.92 (2.53 to 5.30)	<.0001	-2.52 (-3.41 to -1.62)	<.0001
Pancreas	11.76 (10.25 to 13.27)	<.0001	7.45 (5.70 to 9.20)	<.0001	-5.58 (-6.68 to -4.48)	<.0001
Prostate	0.43 (-1.07 to 1.92)	0.576	1.95 (0.22 to 3.68)	0.027	-0.44 (-1.59 to 0.71)	0.452
SCL	7.49 (6.01 to 8.98)	<.0001	6.27 (4.61 to 7.93)	<.0001	-2.74 (-3.82 to -1.66)	<.0001
Age Group At Diagnosis						
66-74 y	Ref		Ref		Ref	
75-84 y	3.54 (2.92 to 4.16)	<.0001	0.55 (-0.17 to 1.27)	0.132	1.69 (1.23 to 2.14)	<.0001
≥85 y	5.72 (4.78 to 6.67)	<.0001	1.13 (0.02 to 2.25)	0.046	4.08 (3.31 to 4.85)	<.0001
Sex						
Male	Ref		Ref		Ref	
Female	5.34 (4.67 to 6.01)	<.0001	2.37 (1.60 to 3.14)	<.0001	-0.90 (-1.40 to -0.40)	<.0001
Race and Ethnicity						
Hispanic	-1.34 (-2.47 to -0.21)	0.02	4.48 (3.18 to 5.79)	<.0001	-4.35 (-4.96 to -3.73)	<.0001
Non-Hispanic Black	-6.99 (-8.06 to -5.91)	<.0001	-2.46 (-3.68 to -1.24)	<.0001	-0.50 (-1.22 to 0.22)	0.173
Non-Hispanic White	Ref		Ref		Ref	
Other [a]	-9.31 (-10.74 to -7.88)	<.0001	0.51 (-1.30 to 2.33)	0.579	-0.93 (-1.89 to 0.04)	0.06
Unknown	-2.62 (-12.02 to 6.78)	0.585	-2.55 (-15.49 to 10.39)	0.7	-6.09 (-10.25 to -1.92)	0.004
Metropolitan Residency [b]						
Non-metropolitan	Ref		Ref		Ref	
Metropolitan	0.16 (-0.88 to 1.20)	0.767	-3.32 (-4.43 to -2.21)	<.0001	-0.22 (-0.95 to 0.50)	0.543
Marital Status						
Married	Ref		Ref		Ref	
Not married	0.45 (-0.34 to 1.24)	0.266	-6.49 (-7.44 to -5.54)	<.0001	3.89 (3.30 to 4.47)	<.0001

© 2026 Hu X et al. *JAMA Network Open*.

	Any Hospice In the Last Year of Life		Home Hospice In the Last Year of Life		Nursing Home Hospice In the Last Year of Life	
	Adjusted Probability Difference (95%CI)	P-value	Adjusted Probability Difference (95%CI)	P-value	Adjusted Probability Difference (95%CI)	P-value
Unknown	0.40 (-1.33 to 2.13)	0.65	-1.60 (-3.62 to 0.42)	0.12	2.77 (1.61 to 3.94)	<.0001
Yost Quintile [c]						
First (Lowest SES)	Ref		Ref		Ref	
Second	0.30 (-0.75 to 1.36)	0.571	0.33 (-0.85 to 1.51)	0.584	0.52 (-0.21 to 1.25)	0.162
Third	1.25 (0.19 to 2.30)	0.02	-0.28 (-1.47 to 0.91)	0.643	0.09 (-0.64 to 0.83)	0.806
Fourth	3.15 (2.09 to 4.20)	<.0001	-0.08 (-1.29 to 1.13)	0.898	-0.23 (-0.98 to 0.52)	0.545
Fifth (Lowest SES)	2.52 (1.46 to 3.59)	<.0001	0.92 (-0.30 to 2.15)	0.14	-1.14 (-1.89 to -0.38)	0.003
Unknown	0.74 (-2.92 to 4.41)	0.691	-2.37 (-6.88 to 2.14)	0.303	0.79 (-1.89 to 3.46)	0.565
Dual Medicare Medicaid Eligibility[d]						
Never Dual	Ref		Ref		Ref	
Gain Dual	1.46 (-0.38 to 3.29)	0.121	-33.51 (-35.97 to -31.06)	<.0001	34.91 (32.50 to 37.31)	<.0001
Lose Dual	-8.50 (-10.08 to -6.93)	<.0001	-18.50 (-20.72 to -16.27)	<.0001	17.19 (15.29 to 19.09)	<.0001
Always Dual	-4.50 (-5.46 to -3.54)	<.0001	-17.18 (-18.43 to -15.93)	<.0001	17.39 (16.30 to 18.48)	<.0001
Other	-2.30 (-4.86 to 0.27)	0.079	-21.89 (-25.24 to -18.54)	<.0001	21.91 (18.91 to 24.92)	<.0001
Medicare Advantage Penetration Rate	7.13 (4.28 to 9.99)	<.0001	-5.22 (-8.69 to -1.75)	0.003	-1.12 (-3.33 to 1.08)	0.317

* *Notes*: MA=Medicare Advantage; NSCL = Non-Small Cell Lung; SCLC = Small Cell Lung; SES = Socioeconomic Status; TM=Traditional Medicare. Regression model also controls for year of death and residence state.

[a] Other group includes American Indian/Alaska Native, Asian/Pacific Islander, and other unspecified.

[b] Based on 2013 Rural Urban Continuum Code. Codes 1-3 were classified as metropolitan, and Codes 4-9 were classified as Non-metropolitan.

[c] The Yost index is a composite SES scores for census tracts based on Median household income, Median house value, Median rent, Percent below 150% of federal poverty line, Education Index, Percent working class, and Percent unemployed. Lower quintiles represent the lower SES groups.

[d] With state buy-in (Medicaid) from cancer diagnosis to 12 months after diagnosis.

© 2026 Hu X et al. *JAMA Network Open*.

eTable 5. Medicare Plan Switching and Patient Characteristics Associated with Hospice Enrollment and Place of Last Hospice Stay Among Beneficiaries With Dual Medicaid and Medicare Enrollment

	**Any Hospice In the Last Year of Life**		**Home Hospice In the Last Year of Life**		**Nursing Home Hospice In the Last Year of Life**	
	**Adjusted Probability Difference (95%CI)**	**P-value**	**Adjusted Probability Difference (95%CI)**	**P-value**	**Adjusted Probability Difference (95%CI)**	**P-value**
**Total N**	39098		25291		25291	
Medicare Plan Switching						
Full TM	Ref		Ref		Ref	
TM to MA	3.66 (0.45 to 6.87)	0.026	3.13 (-0.96 to 7.22)	0.133	-1.92 (-5.43 to 1.60)	0.285
MA to TM	1.81 (-0.76 to 4.38)	0.168	-7.75 (-11.20 to -4.30)	<.0001	6.01 (2.80 to 9.21)	<.0001
Full MA	9.15 (8.10 to 10.20)	<.0001	2.71 (1.36 to 4.06)	<.0001	-4.30 (-5.45 to -3.15)	<.0001
Other	1.66 (-3.34 to 6.66)	0.516	3.00 (-3.65 to 9.65)	0.377	-1.45 (-7.35 to 4.46)	0.63
Cancer Site						
Breast	Ref		Ref		Ref	
Colorectal	7.10 (4.95 to 9.24)	<.0001	6.78 (4.07 to 9.49)	<.0001	-4.63 (-7.12 to -2.14)	<.0001
NSCL	3.50 (1.52 to 5.48)	0.001	7.70 (5.22 to 10.19)	<.0001	-6.66 (-8.96 to -4.37)	<.0001
Pancreas	12.49 (10.10 to 14.87)	<.0001	12.77 (9.73 to 15.82)	<.0001	-12.52 (-15.22 to -9.83)	<.0001
Prostate	-0.95 (-3.52 to 1.63)	0.47	1.86 (-1.48 to 5.20)	0.275	-1.10 (-4.17 to 1.98)	0.486
SCL	6.13 (3.79 to 8.48)	<.0001	11.74 (8.84 to 14.65)	<.0001	-10.23 (-12.83 to -7.63)	<.0001
Age Group At Diagnosis						
66-74 y	Ref		Ref		Ref	
75-84 y	2.09 (1.09 to 3.09)	<.0001	0.72 (-0.57 to 2.00)	0.275	1.63 (0.52 to 2.74)	0.004
≥85 y	3.36 (1.84 to 4.88)	<.0001	0.73 (-1.26 to 2.73)	0.471	4.57 (2.80 to 6.33)	<.0001
Sex						
Male	Ref		Ref		Ref	
Female	4.96 (3.92 to 6.01)	<.0001	5.35 (4.02 to 6.67)	<.0001	-4.04 (-5.20 to -2.87)	<.0001
Race and Ethnicity						
Hispanic	-2.21 (-3.61 to -0.81)	0.002	12.70 (10.89 to 14.51)	<.0001	-12.81 (-14.20 to -11.42)	<.0001
Non-Hispanic Black	-7.06 (-8.42 to -5.70)	<.0001	0.85 (-0.89 to 2.58)	0.338	-2.13 (-3.68 to -0.59)	0.007
Non-Hispanic White	Ref		Ref		Ref	
Other [a]	-9.60 (-11.27 to -7.92)	<.0001	4.97 (2.64 to 7.30)	<.0001	-5.83 (-7.76 to -3.90)	<.0001
Unknown	0.42 (-11.29 to 12.14)	0.944	4.30 (-12.83 to 21.44)	0.623	-15.11 (-25.46 to -4.75)	0.004
Metropolitan Residency [b]						
Non-metropolitan	Ref		Ref		Ref	
Metropolitan	-0.33 (-2.00 to 1.33)	0.693	-3.63 (-5.62 to -1.64)	<.0001	0.88 (-0.84 to 2.60)	0.317
Marital Status						
Married	Ref		Ref		Ref	

© 2026 Hu X et al. *JAMA Network Open*.

Not married	-0.40 (-1.75 to 0.94)	0.559	-11.34 (-13.09 to -9.59)	<.0001	9.07 (7.69 to 10.44)	<.0001
Unknown	-0.05 (-2.92 to 2.83)	0.974	-9.14 (-12.87 to -5.42)	<.0001	10.21 (7.36 to 13.07)	<.0001
Yost Quintile[c]						
First (Lowest SES)	Ref		Ref		Ref	
Second	-0.70 (-2.03 to 0.64)	0.307	0.35 (-1.34 to 2.05)	0.684	0.62 (-0.87 to 2.11)	0.416
Third	0.53 (-0.91 to 1.97)	0.469	-0.05 (-1.89 to 1.79)	0.959	-0.08 (-1.67 to 1.51)	0.921
Fourth	1.52 (0.00 to 3.03)	0.049	-1.54 (-3.52 to 0.44)	0.128	0.48 (-1.22 to 2.18)	0.58
Fifth (Lowest SES)	-0.27 (-1.92 to 1.39)	0.754	-0.86 (-3.06 to 1.34)	0.443	-1.00 (-2.84 to 0.83)	0.282
Unknown	2.38 (-1.53 to 6.29)	0.233	-6.73 (-12.30 to -1.15)	0.018	1.37 (-3.78 to 6.52)	0.602
Medicare Advantage Penetration Rate	0.05 (-4.56 to 4.67)	0.982	-8.18 (-14.24 to -2.12)	0.008	-2.13 (-7.35 to 3.09)	0.424

* *Notes*: MA=Medicare Advantage; NSCL = Non-Small Cell Lung; SCLC = Small Cell Lung; TM=Traditional Medicare; SES = Socioeconomic Status. Regression model also controls for year of death and residence state.

[a] Other group includes American Indian/Alaska Native, Asian/Pacific Islander, and other unspecified.

[b] Based on 2013 Rural Urban Continuum Code. Codes 1-3 were classified as metropolitan, and Codes 4-9 were classified as Non-metropolitan.

[c] The Yost index is a composite SES scores for census tracts based on Median household income, Median house value, Median rent, Percent below 150% of federal poverty line, Education Index, Percent working class, and Percent unemployed. Lower quintiles represent the lower SES groups.

© 2026 Hu X et al. *JAMA Network Open*.

eTable 6. Medicare Plan Switching and Patient Characteristics Associated with Hospice Enrollment and Place of Last Hospice Stay Among Beneficiaries Without Dual Medicaid and Medicare Enrollment

	Any Hospice In the Last Year of Life		Home Hospice In the Last Year of Life		Nursing Home Hospice In the Last Year of Life	
	**Adjusted Probability Difference (95%CI)**	**P-value**	**Adjusted Probability Difference (95%CI)**	**P-value**	**Adjusted Probability Difference (95%CI)**	**P-value**
**Total N**	155185		111575		111575	
Medicare Plan Switching						
Full TM	Ref		Ref		Ref	
TM to MA	2.08 (0.15 to 4.01)	0.034	1.16 (-1.11 to 3.43)	0.315	-0.45 (-1.74 to 0.84)	0.494
MA to TM	3.92 (2.03 to 5.81)	<.0001	-1.79 (-3.91 to 0.33)	0.097	1.27 (0.16 to 2.37)	0.025
Full MA	6.32 (5.81 to 6.84)	<.0001	1.63 (1.05 to 2.21)	<.0001	-1.12 (-1.45 to -0.79)	<.0001
Other	3.43 (-1.84 to 8.70)	0.202	1.17 (-5.01 to 7.35)	0.711	1.27 (-1.91 to 4.45)	0.435
Cancer Site						
Breast	Ref		Ref		Ref	
Colorectal	5.99 (4.82 to 7.16)	<.0001	3.02 (1.71 to 4.33)	<.0001	-1.07 (-1.72 to -0.41)	0.001
NSCL	2.44 (1.38 to 3.49)	<.0001	2.34 (1.15 to 3.53)	<.0001	-1.41 (-2.00 to -0.82)	<.0001
Pancreas	11.80 (10.58 to 13.02)	<.0001	5.96 (4.61 to 7.32)	<.0001	-3.24 (-3.96 to -2.52)	<.0001
Prostate	0.37 (-0.91 to 1.66)	0.571	0.81 (-0.70 to 2.32)	0.292	0.52 (-0.21 to 1.26)	0.164
SCL	5.37 (4.16 to 6.59)	<.0001	3.94 (2.58 to 5.29)	<.0001	-1.51 (-2.21 to -0.82)	<.0001
Age Group At Diagnosis						
66-74 y	Ref		Ref		Ref	
75-84 y	5.24 (4.77 to 5.71)	<.0001	0.94 (0.39 to 1.48)	0.001	1.69 (1.38 to 1.99)	<.0001
≥85 y	10.53 (9.79 to 11.27)	<.0001	3.33 (2.51 to 4.16)	<.0001	3.22 (2.83 to 3.62)	<.0001
Sex						
Male	Ref		Ref		Ref	
Female	6.32 (5.84 to 6.81)	<.0001	1.04 (0.48 to 1.59)	<.0001	0.25 (-0.06 to 0.56)	0.113
Race and Ethnicity						
Hispanic	-2.56 (-3.56 to -1.57)	<.0001	2.53 (1.26 to 3.80)	<.0001	-2.49 (-3.25 to -1.72)	<.0001
Non-Hispanic Black	-9.08 (-9.88 to -8.27)	<.0001	-2.55 (-3.54 to -1.55)	<.0001	-0.53 (-1.13 to 0.07)	0.083
Non-Hispanic White	Ref		Ref		Ref	
Other [a]	-9.19 (-10.39 to -7.99)	<.0001	-1.77 (-3.55 to 0.00)	0.051	0.36 (-0.57 to 1.29)	0.446
Unknown	-4.32 (-12.70 to 4.06)	0.313	1.73 (-11.65 to 15.11)	0.8	-7.00 (-17.65 to 3.65)	0.198
Metropolitan Residency [b]						
Non-metropolitan	Ref		Ref		Ref	
Metropolitan	0.90 (0.11 to 1.68)	0.025	-4.15 (-5.03 to -3.27)	<.0001	-0.67 (-1.12 to -0.21)	0.004
Marital Status						
Married	Ref					

© 2026 Hu X et al. *JAMA Network Open*.

Not married	0.78 (0.18 to 1.39)	0.011	-4.44 (-5.14 to -3.75)	<.0001	2.74 (2.36 to 3.12)	<.0001
Unknown	-0.63 (-1.98 to 0.72)	0.358	0.30 (-1.34 to 1.94)	0.717	1.34 (0.45 to 2.24)	0.003
Yost Quintile [c]						
First (Lowest SES)	Ref		Ref		Ref	
Second	1.02 (0.19 to 1.85)	0.016	0.72 (-0.23 to 1.67)	0.139	0.21 (-0.31 to 0.73)	0.431
Third	1.71 (0.89 to 2.54)	<.0001	-0.44 (-1.38 to 0.49)	0.353	0.05 (-0.47 to 0.57)	0.847
Fourth	2.74 (1.91 to 3.57)	<.0001	-0.17 (-1.11 to 0.78)	0.731	-0.18 (-0.71 to 0.34)	0.497
Fifth (Lowest SES)	2.24 (1.40 to 3.07)	<.0001	0.29 (-0.67 to 1.25)	0.557	-0.66 (-1.20 to -0.12)	0.016
Unknown	-0.06 (-3.27 to 3.16)	0.973	-4.86 (-8.55 to -1.17)	0.01	1.47 (-0.43 to 3.37)	0.129
Medicare Advantage Penetration Rate	5.72 (3.55 to 7.88)	<.0001	-9.06 (-11.71 to -6.41)	<.0001	1.19 (-0.28 to 2.65)	0.113

* *Notes*: MA=Medicare Advantage; NSCL = Non-Small Cell Lung; SCL = Small Cell Lung; SES = Socioeconomic Status; TM=Traditional Medicare. Regression model also controls for year of death and residence state.

[a] Other group includes American Indian/Alaska Native, Asian/Pacific Islander, and other unspecified.

[b] Based on 2013 Rural Urban Continuum Code. Codes 1-3 were classified as metropolitan, and Codes 4-9 were classified as Non-metropolitan.

[c] The Yost index is a composite SES scores for census tracts based on Median household income, Median house value, Median rent, Percent below 150% of federal poverty line, Education Index, Percent working class, and Percent unemployed.

© 2026 Hu X et al. *JAMA Network Open*.

eTable 7. Association Between Medicare Plan Switching and Hospice Enrollment and Place of Last Hospice Stay With and Without Adjusting for NCI Comorbidity Index

	Any Hospice In the Last Year of Life				Nursing Home Hospice In the Last Year of Life			
	No Control for Comorbidity		Control for Comorbidity		No Control for Comorbidity		Control for Comorbidity	
	**Adjusted Probability Difference (95%CI)**	**P-value**	**Adjusted Probability Difference (95%CI)**	**P-value**	**Adjusted Probability Difference (95%CI)**	**P-value**	**Adjusted Probability Difference (95%CI)**	**P-value**
**Total N**	71,305		71,305		50,457		50,457	
Medicare Plan Switching								
Full TM	Ref		Ref		Ref		Ref	
TM to MA	2.17 (-0.38 to 4.72)	0.096	2.08 (-0.47 to 4.63)	0.11	-0.43 (-2.27 to 1.40)	0.644	-0.37 (-2.21 to 1.48)	0.698
MA to TM	1.90 (-0.45 to 4.24)	0.113	2.13 (-0.21 to 4.46)	0.074	4.00 (1.99 to 6.01)	<.0001	3.89 (1.89 to 5.88)	<.0001
Full MA	6.41 (5.70 to 7.11)	<.0001	6.43 (5.73 to 7.14)	<.0001	-1.66 (-2.17 to -1.15)	<.0001	-1.67 (-2.18 to -1.16)	<.0001
Other	2.41 (-3.06 to 7.88)	0.387	2.31 (-3.16 to 7.79)	0.407	0.47 (-3.27 to 4.21)	0.804	0.46 (-3.26 to 4.19)	0.807
Cancer Site								
Breast	Ref		Ref		Ref		Ref	
Colorectal	6.80 (5.01 to 8.58)	<.0001	6.66 (4.88 to 8.44)	<.0001	-1.54 (-2.83 to -0.26)	0.018	-1.51 (-2.81 to -0.21)	0.023
NSCL	1.85 (0.19 to 3.51)	0.029	2.05 (0.39 to 3.71)	0.016	-1.96 (-3.15 to -0.77)	0.001	-2.19 (-3.39 to -0.98)	<.0001
Pancreas	12.54 (10.77 to 14.31)	<.0001	12.55 (10.77 to 14.32)	<.0001	-4.31 (-5.59 to -3.02)	<.0001	-4.42 (-5.72 to -3.13)	<.0001
Prostate	0.97 (-1.04 to 2.98)	0.344	0.93 (-1.08 to 2.94)	0.363	-0.12 (-1.64 to 1.39)	0.872	-0.10 (-1.63 to 1.43)	0.898
SCL	6.71 (4.87 to 8.56)	<.0001	7.13 (5.28 to 8.97)	<.0001	-2.24 (-3.57 to -0.91)	0.001	-2.62 (-3.96 to -1.29)	<.0001
Age Group At Diagnosis								
66-74 y	Ref		Ref		Ref		Ref	
75-84 y	5.40 (4.69 to 6.11)	<.0001	5.78 (5.07 to 6.49)	<.0001	1.46 (0.94 to 1.98)	<.0001	1.27 (0.75 to 1.79)	<.0001
≥85 y	10.32 (9.35 to 11.29)	<.0001	10.76 (9.80 to 11.72)	<.0001	3.27 (2.48 to 4.05)	<.0001	3.04 (2.26 to 3.82)	<.0001
Sex								
Male	Ref		Ref		Ref		Ref	
Female	6.19 (5.47 to 6.91)	<.0001	5.88 (5.16 to 6.60)	<.0001	-0.97 (-1.50 to -0.43)	<.0001	-0.81 (-1.34 to -0.27)	0.003
Race and Ethnicity								
Hispanic	-3.06 (-4.34 to -1.78)	<.0001	-3.14 (-4.42 to -1.86)	<.0001	-4.46 (-5.14 to -3.78)	<.0001	-4.39 (-5.08 to -3.71)	<.0001
Non-Hispanic Black	-10.24 (-11.45 to -9.03)	<.0001	-9.90 (-11.11 to -8.70)	<.0001	-0.70 (-1.51 to 0.10)	0.085	-0.80 (-1.59 to -0.01)	0.048
Non-Hispanic White	Ref		Ref		Ref		Ref	
Other [a]	-10.07 (-11.69 to -8.45)	<.0001	-10.38 (-12.00 to -8.75)	<.0001	-1.48 (-2.56 to -0.41)	0.007	-1.30 (-2.39 to -0.21)	0.019
Unknown	-1.46 (-10.75 to 7.83)	0.759	-1.73 (-11.04 to 7.59)	0.716	-5.23 (-10.19 to -0.28)	0.039	-4.92 (-10.16 to 0.32)	0.066
Metropolitan Residency [b]								
Non-metropolitan	Ref		Ref		Ref		Ref	
Metropolitan	1.02 (-0.17 to 2.21)	0.092	1.17 (-0.01 to 2.36)	0.053	-0.38 (-1.21 to 0.45)	0.366	-0.45 (-1.28 to 0.38)	0.292
Marital Status								
Married	Ref		Ref		Ref		Ref	
Not married	1.11 (0.23 to 1.99)	0.014	1.15 (0.27 to 2.03)	0.01	4.45 (3.79 to 5.12)	<.0001	4.44 (3.78 to 5.10)	<.0001
Unknown	-0.67 (-2.75 to 1.42)	0.53	-0.79 (-2.87 to 1.29)	0.455	3.50 (2.13 to 4.86)	<.0001	3.52 (2.15 to 4.88)	<.0001
Yost Quintile [c]								
First (Lowest SES)	Ref		Ref		Ref		Ref	
Second	-0.05 (-1.22 to 1.11)	0.927	-0.05 (-1.21 to 1.10)	0.928	0.89 (0.07 to 1.71)	0.033	0.90 (0.08 to 1.71)	0.031

© 2026 Hu X et al. *JAMA Network Open*.

Third	0.42 (-0.75 to 1.58)	0.483	0.38 (-0.79 to 1.54)	0.526	-0.20 (-1.02 to 0.61)	0.624	-0.17 (-0.98 to 0.64)	0.683
Fourth	1.15 (-0.03 to 2.33)	0.056	1.09 (-0.08 to 2.26)	0.069	0.08 (-0.76 to 0.92)	0.845	0.12 (-0.72 to 0.95)	0.785
Fifth (Lowest SES)	1.44 (0.25 to 2.64)	0.017	1.30 (0.11 to 2.49)	0.032	-1.21 (-2.05 to -0.37)	0.005	-1.12 (-1.96 to -0.29)	0.009
Unknown	0.59 (-3.19 to 4.36)	0.761	0.54 (-3.23 to 4.31)	0.779	2.05 (-0.94 to 5.04)	0.178	2.08 (-0.90 to 5.07)	0.171
Medicare Advantage Penetration Rate	1.69 (-1.42 to 4.80)	0.286	1.81 (-1.30 to 4.91)	0.253	0.76 (-1.65 to 3.18)	0.535	0.56 (-1.85 to 2.97)	0.647
NCI Comorbidity Index								
0			Ref				Ref	
0.1-1			1.26 (0.36 to 2.16)	0.006			1.16 (0.51 to 1.80)	<.0001
> 1			-4.28 (-5.22 to -3.34)	<.0001			3.12 (2.45 to 3.80)	<.0001

* *Notes*: MA=Medicare Advantage; NSCL = Non-Small Cell Lung; SCL = Small Cell Lung; SES = Socioeconomic Status; TM=Traditional Medicare. Regression model also controls for year of death and residence state.

[a] Other group includes American Indian/Alaska Native, Asian/Pacific Islander, and other unspecified.

[b] Based on 2013 Rural Urban Continuum Code. Codes 1-3 were classified as metropolitan, and Codes 4-9 were classified as Non-metropolitan.

[c] The Yost index is a composite SES scores for census tracts based on Median household income, Median house value, Median rent, Percent below 150% of federal poverty line, Education Index, Percent working class, and Percent unemployed. Lower quintiles represent the lower SES groups.

© 2026 Hu X et al. *JAMA Network Open*.

eTable 8. Medicare Plan Switching Stratified by Skilled Nursing Facility Stay Post Cancer Diagnosis

	Continuous TM	Continuous MA	TM to MA	MA to TM	Other	Total
**Total N, Row %**	47766 (62.2)	26193 (34.1)	1165 (1.5)	1458 (1.9)	247 (0.3)	76829 (100.0)
**SNF Post Cancer Diagnosis**						
**No**	34607 (60.5)	20728 (36.2)	836 (1.5)	863 (1.5)	159 (0.3)	57193 (100.0)
**Yes**	13159 (67.0)	5465 (27.8)	329 (1.7)	595 (3.0)	88 (0.5)	19636 (100.0)

* *Notes*: MA=Medicare Advantage; SNF=Skilled Nursing Facility; TM=Traditional Medicare.

© 2026 Hu X et al. *JAMA Network Open*.

eTable 9. Medicare Plan Switching and Patient Characteristics Associated with Nursing Home Hospice Enrollment With and Without Adjusting for Skilled Nursing Facility Stay Post Cancer Diagnosis

	**Any Hospice In the Last Year of Life**				**Any Nursing Home Hospice In the Last Year of Life**			
	No Control for SNF		Control for SNF		No control for SNF		Control for SNF	
	**Adjusted Probability Difference (95%CI)**	**P-value**	**Adjusted Probability Difference (95%CI)**	**P-value**	**Adjusted Probability Difference (95%CI)**	**P-value**	**Adjusted Probability Difference (95%CI)**	**P-value**
**Total N**	76829		76829		54600		54600	
Medicare Plan Switching								
Full TM	Ref		Ref		Ref		Ref	
TM to MA	3.30 (0.79 to 5.81)	0.01	3.23 (0.73 to 5.73)	0.011	0.03 (-1.85 to 1.91)	0.972	0.29 (-1.52 to 2.09)	0.756
MA to TM	2.38 (0.15 to 4.62)	0.037	2.94 (0.73 to 5.15)	0.009	3.54 (1.62 to 5.45)	<.0001	2.38 (0.66 to 4.10)	0.007
Full MA	6.78 (6.10 to 7.46)	<.0001	6.50 (5.81 to 7.18)	<.0001	-1.75 (-2.26 to -1.24)	<.0001	-0.90 (-1.41 to -0.40)	<.0001
Other	0.97 (-4.44 to 6.38)	0.726	1.23 (-4.15 to 6.61)	0.654	-1.51 (-5.02 to 2.01)	0.401	-1.73 (-4.96 to 1.49)	0.292
Cancer Site								
Breast	Ref		Ref		Ref		Ref	
Colorectal	6.25 (4.48 to 8.01)	<.0001	6.05 (4.30 to 7.80)	<.0001	-1.66 (-2.97 to -0.36)	0.013	-1.24 (-2.42 to -0.07)	0.038
NSCL	2.18 (0.54 to 3.82)	0.009	1.78 (0.15 to 3.41)	0.033	-2.23 (-3.44 to -1.01)	<.0001	-1.10 (-2.19 to 0.00)	0.049
Pancreas	11.80 (10.04 to 13.55)	<.0001	10.95 (9.20 to 12.71)	<.0001	-4.82 (-6.12 to -3.52)	<.0001	-2.43 (-3.66 to -1.20)	<.0001
Prostate	1.06 (-0.94 to 3.05)	0.299	1.25 (-0.73 to 3.23)	0.217	0.14 (-1.41 to 1.69)	0.857	-0.45 (-1.82 to 0.91)	0.518
SCL	6.08 (4.26 to 7.89)	<.0001	5.58 (3.78 to 7.39)	<.0001	-3.16 (-4.50 to -1.83)	<.0001	-1.71 (-2.94 to -0.48)	0.006
Age Group At Diagnosis								
66-74 y	Ref		Ref		Ref		Ref	
75-84 y	5.30 (4.62 to 5.98)	<.0001	5.54 (4.86 to 6.23)	<.0001	1.22 (0.71 to 1.72)	<.0001	0.78 (0.29 to 1.27)	0.002
≥85 y	10.17 (9.24 to 11.10)	<.0001	10.47 (9.54 to 11.40)	<.0001	3.29 (2.52 to 4.06)	<.0001	2.65 (1.92 to 3.39)	<.0001
Sex								
Male	Ref		Ref		Ref		Ref	
Female	6.02 (5.33 to 6.70)	<.0001	6.15 (5.46 to 6.83)	<.0001	-1.09 (-1.61 to -0.56)	<.0001	-1.29 (-1.80 to -0.79)	<.0001
Race and Ethnicity								
Hispanic	-2.99 (-4.23 to -1.74)	<.0001	-3.31 (-4.56 to -2.06)	<.0001	-4.50 (-5.18 to -3.82)	<.0001	-3.61 (-4.32 to -2.90)	<.0001
Non-Hispanic Black	-9.77 (-10.94 to -8.60)	<.0001	-9.60 (-10.77 to -8.44)	<.0001	-0.99 (-1.78 to -0.20)	0.014	-1.18 (-1.92 to -0.43)	0.002
Non-Hispanic White	Ref		Ref		Ref		Ref	
Other [a]	-9.92 (-11.51 to -8.33)	<.0001	-10.19 (-11.78 to -8.60)	<.0001	-1.51 (-2.57 to -0.45)	0.005	-0.72 (-1.80 to 0.36)	0.192
Unknown	-8.46 (-20.71 to 3.78)	0.175	-8.53 (-20.79 to 3.72)	0.172	-5.65 (-11.83 to 0.53)	0.073	-5.00 (-11.64 to 1.64)	0.14
Metropolitan Residency [b]								
Non-metropolitan	Ref		Ref		Ref		Ref	
Metropolitan	0.34 (-0.79 to 1.47)	0.56	0.47 (-0.66 to 1.60)	0.412	-0.53 (-1.35 to 0.29)	0.207	-0.76 (-1.57 to 0.04)	0.064
Marital Status								
Married	Ref		Ref		Ref		Ref	
Not married	0.78 (-0.06 to 1.63)	0.07	1.16 (0.32 to 2.01)	0.007	4.34 (3.69 to 4.99)	<.0001	3.46 (2.86 to 4.07)	<.0001
Unknown	-1.22 (-3.23 to 0.79)	0.233	-1.22 (-3.24 to 0.79)	0.233	3.44 (2.08 to 4.81)	<.0001	3.47 (2.11 to 4.84)	<.0001
Yost Quintile [c]								
First (Lowest SES)	Ref		Ref		Ref		Ref	
Second	0.24 (-0.88 to 1.36)	0.676	0.24 (-0.88 to 1.35)	0.678	1.05 (0.25 to 1.85)	0.01	1.22 (0.45 to 1.99)	0.002
Third	1.20 (0.08 to 2.32)	0.036	1.21 (0.09 to 2.32)	0.035	0.11 (-0.68 to 0.91)	0.784	0.13 (-0.64 to 0.89)	0.747

© 2026 Hu X et al. *JAMA Network Open*.

	Any Hospice In the Last Year of Life				Any Nursing Home Hospice In the Last Year of Life			
	No Control for SNF		Control for SNF		No control for SNF		Control for SNF	
	**Adjusted Probability Difference (95%CI)**	**P-value**	**Adjusted Probability Difference (95%CI)**	**P-value**	**Adjusted Probability Difference (95%CI)**	**P-value**	**Adjusted Probability Difference (95%CI)**	**P-value**
**Total N**	76829		76829		54600		54600	
Fourth	1.73 (0.60 to 2.86)	0.003	1.72 (0.59 to 2.85)	0.003	0.33 (-0.49 to 1.16)	0.425	0.35 (-0.44 to 1.14)	0.389
Fifth (Lowest SES)	2.10 (0.95 to 3.24)	<.0001	2.06 (0.92 to 3.21)	<.0001	-0.80 (-1.63 to 0.03)	0.059	-0.67 (-1.47 to 0.13)	0.099
Unknown	0.67 (-3.20 to 4.54)	0.734	0.62 (-3.25 to 4.49)	0.753	2.29 (-0.81 to 5.40)	0.148	2.14 (-0.81 to 5.09)	0.156
Dual Medicare Medicaid Eligibility Change [d]								
Never Dual	Ref		Ref		Ref		Ref	
Gain Dual	2.00 (-0.04 to 4.04)	0.055	3.35 (1.36 to 5.34)	0.001	32.53 (29.84 to 35.23)	<.0001	25.18 (22.79 to 27.58)	<.0001
Lose Dual	-5.29 (-6.90 to -3.68)	<.0001	-4.82 (-6.42 to -3.22)	<.0001	21.26 (19.01 to 23.51)	<.0001	18.54 (16.50 to 20.57)	<.0001
Always Dual	-2.23 (-3.26 to -1.19)	<.0001	-1.71 (-2.74 to -0.69)	0.001	16.21 (15.07 to 17.36)	<.0001	14.20 (13.15 to 15.24)	<.0001
Other	-1.63 (-4.61 to 1.36)	0.286	-0.61 (-3.55 to 2.32)	0.682	22.74 (18.99 to 26.48)	<.0001	17.30 (14.12 to 20.49)	<.0001
Medicare Advantage Penetration Rate	2.93 (-0.10 to 5.96)	0.058	2.45 (-0.58 to 5.48)	0.112	0.60 (-1.80 to 3.00)	0.623	0.83 (-1.49 to 3.15)	0.484
SNF Post Cancer Diagnosis								
No			Ref				Ref	
Yes			-5.49 (-6.25 to -4.72)	<.0001			14.22 (13.53 to 14.91)	<.0001

* *Notes*: MA=Medicare Advantage; NSCL = Non-Small Cell Lung; SCL = Small Cell Lung; SES = Socioeconomic Status; SNF=Skilled nursing facility; TM=Traditional Medicare. Regression model also controls for year of death and residence state.

[a] Other group includes American Indian/Alaska Native, Asian/Pacific Islander, and other unspecified.

[b] Based on 2013 Rural Urban Continuum Code. Codes 1-3 were classified as metropolitan, and Codes 4-9 were classified as Non-metropolitan.

[c] The Yost index is a composite SES scores for census tracts based on Median household income, Median house value, Median rent, Percent below 150% of federal poverty line, Education Index, Percent working class, and Percent unemployed. Lower quintiles represent the lower SES groups.

[d] With state buy-in (Medicaid) from cancer diagnosis to the end of follow-up.

© 2026 Hu X et al. *JAMA Network Open*.
