# Supplementary material for: Medicare Plan Switching and Hospice Care Among Decedents With Advanced Cancer
Source: JAMA Netw Open. 2026 Mar 24;9(3):e260755. doi: 10.1001/jamanetworkopen.2026.0755 (PMC13014202; doi:10.1001/jamanetworkopen.2026.0755)
Supplement: Supplement 2. — Data Sharing Statement [file jamanetwopen-e260755-s002.pdf]

Data Sharing Statement

Hu. Medicare Plan Switching and Hospice Care Among Decedents With Advanced Cancer. *JAMA Netw Open*. Published March 24, 2026. doi:10.1001/jamanetworkopen.2026.0755

Data
Data available: No
